# Physics‐Informed Machine Learning for Sustainable Alloy Design: Toward a Recyclable Unified Q&P Steel

**DOI:** 10.1002/advs.75515

**Published:** 2026-06-15

**Authors:** Xiaolu Wei, Yong Li, Chenchong Wang, Lingyu Wang, Xiang Song, Keming Mao, Yu Zhang, Wei Xu

**Affiliations:** ^1^ The State Key Laboratory of Digital Steel Northeastern University Shenyang China; ^2^ Software College Northeastern University Shenyang China; ^3^ Ansteel Beijing Research Institute Co., Ltd. Beijing China

**Keywords:** physics‐informed machine learning, property bridging, quenching and partitioning steels, sustainable alloy design, unified‐composition steels

## Abstract

For sustainable alloy design, unified‐composition approaches offer an effective route to deliver multiple performance levels while reducing chemistry complexity. Quenching and partitioning (Q&P) steels are widely used advanced high‐strength steels, yet their grade development typically relies on distinct, grade‐specific chemistries, complicating welding and hindering efficient recycling. This study proposes a physics‐informed machine learning framework for unified‐composition Q&P steel design, enabling multiple strength grades from a single alloy via heat‐treatment tuning. A physics‐guided property‐bridging model integrates metallurgical descriptors with near‐high‐throughput hardness data, transferring knowledge from hardness to tensile properties under sparse tensile labels. This approach enables improved prediction of tensile strength and elongation from limited tensile datasets. A multi‐objective genetic algorithm then explores the composition‐process space to identify one alloy that meets the Q&P980, Q&P1180, and Q&P1380 grade targets via processing adjustments. Compared to a purely data‐driven baseline, the framework substantially improves tensile prediction accuracy (R^2^ up to 84% vs. 74%) while maintaining better model stability under data‐sparse conditions. An experimental validation demonstrates that the same chemistry can achieve ∼980, ∼1180, and ∼1380 MPa tensile strengths with suitable ductility under different Q&P schedules. Overall, the physics‐informed framework exemplifies a paradigm for sustainable alloy design that reduces chemistry variants while simplifying recycling.

## Graphical abstract

A physics‐informed property‐bridging framework links high‐throughput hardness screening to tensile performance in quenching and partitioning steels. By transferring metallurgically guided representations across properties, a single alloy composition is designed to achieve multiple strength grades through heat‐treatment tuning alone, offering a sustainable pathway to reduce chemistry proliferation and improve steel recyclability.

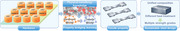

## 1 Introduction

Quenching and partitioning (Q&P) steels represent a key class of third‐generation advanced high‐strength steels, where a tailored martensite/retained‐austenite microstructure provides an excellent strength‐ductility balance and supports widespread use in automotive body structures and other crash‐relevant components [1, 2]. In the Q&P process, a steel is first partially quenched to form martensite and then “partitioned” at an intermediate temperature to allow carbon to diffuse from supersaturated martensite into the remaining austenite, thereby stabilizing that austenite against further transformation [3, 4]. The resulting mixture of martensite and retained austenite (RA) provides high strength together with transformation‐induced plasticity (TRIP)‐assisted ductility. Since the 2010s, Q&P steels with improved formability and crash energy absorption have been widely adopted as representative third‐generation advanced high‐strength steels (AHSS) in car bodies [5, 6].

Despite these advantages, Q&P steels are typically developed as separate grades (e.g., 980, 1180, 1380 MPa tensile strength), each with a distinct composition tuned to its target strength level. This multi‐steel strategy creates substantial challenges for both manufacturing and sustainability. The proliferation of different chemistries complicates welding, owing to differences in hardenability and electrical resistivity, and it severely hinders efficient recycling, since scrap must be sorted by alloy type to avoid off‐grade contamination of secondary steels [7, 8, 9, 10]. As the steel industry is increasingly driven by decarbonization and circular‐economy targets, such chemical fragmentation becomes a significant barrier to low‐carbon production and high‐value recycling [11]. In this context, the idea of a “unified‐composition” alloy, where a single alloy chemistry can be processed into multiple strength grades by tailored microstructural control, offers an attractive pathway to reduce alloy diversity, simplify joining, and enable closed‐loop scrap utilization.

Recent works have provided compelling proof‐of‐concept for this unified‐alloy paradigm [12, 13]. Lu et al. demonstrated a unified steel (“UniSteel”) that spans 600–1680 MPa strength levels by varying heat treatments on a single alloy, greatly simplifying joining and scrap reuse in automotive applications [12]. A unified Q&P steel would be especially impactful: if all Q&P grades could be achieved from one recyclable alloy, it would remove the need to segregate Q&P scrap by grade and could significantly lower the carbon footprint associated with end‐of‐life vehicle recycling. However, designing such a steel is far from trivial. The alloy must be lean and cost‐effective, compatible with conventional steelmaking, and flexible enough to form the diverse microstructures required for different strength‐ductility combinations [12]. Traditional trial‐and‐error development to satisfy such divergent microstructural states would be both time‐consuming and resource‐intensive.

Against this backdrop, materials informatics and artificial intelligence (AI) have emerged as powerful tools for accelerating alloy design [14, 15]. Recent advances in data‐driven modeling have demonstrated the potential of machine‐learning approaches to predict phase stability [16], transformation behavior [17], and strength‐ductility responses [18, 19, 20], thereby enabling more efficient exploration of complex composition‐process‐property spaces [21, 22, 23, 24]. In particular, Ma et al. [25] recently demonstrated a generative model‐guided unified‐composition dual‐phase (DP) steel, in which a single chemical composition spans multiple strength grades through controlled processing. This line of work underscores the promise of AI‐assisted unified‐composition design as a route toward more sustainable structural steels. However, extending the unified‐composition concept from DP to Q&P steels introduces substantially greater metallurgical complexity. Whereas DP steels are characterized primarily by a ferrite‐martensite mixture [26], Q&P steels rely on precise control of carbon partitioning, retained austenite stabilization, and multi‐stage phase transformations during quenching and partitioning [27]. While emerging machine learning approaches increasingly attempt to incorporate domain knowledge [28, 29, 30, 31, 32], explicit and quantitative integration of the metallurgical mechanisms governing Q&P processing remains limited. Most existing models rely on statistical correlations within relatively narrow composition or process windows, which restricts their transferability across the diverse processing paths characteristic of Q&P steels [33, 34]. This gap poses a critical challenge: without physics‐aware representations of the underlying transformations, it is difficult to design unified‐composition Q&P steels that simultaneously and reliably bridge multiple strength‐ductility targets and remain robust to processing variations. From the perspective of AI for sustainable materials, closing this gap is essential for leveraging AI not only as a predictor, but as a design engine that can directly support recycling‐friendly, low‐carbon alloy concepts.

A second, equally important challenge is data scarcity in the relevant design space. To robustly design unified Q&P steels, predictive models must interpolate and extrapolate across variations in composition, austenitization, quench temperature, partitioning schedule, and cooling path. Generating tensile data over such an enlarged space is experimentally prohibitive: each tensile label requires large‐scale sample preparation, machining, and testing. By contrast, small‐sample Q&P treatments combined with hardness measurements can be performed at near‐high throughput, providing a much denser mapping of the composition‐processing space. Hardness, however, is not a direct proxy for full tensile behavior; it captures aspects of strength but only indirectly reflects ductility and the detailed evolution of RA. Naively combining hardness and tensile data, or treating hardness as a simple surrogate label, risks introducing systematic bias or domain shift. This motivates a transfer learning‐based property‐bridging strategy: [35, 36, 37, 38] explicitly learning from rapid‐screening hardness data and then transferring that knowledge, in a physics‐aware manner, to predict tensile properties.

**FIGURE 1:**
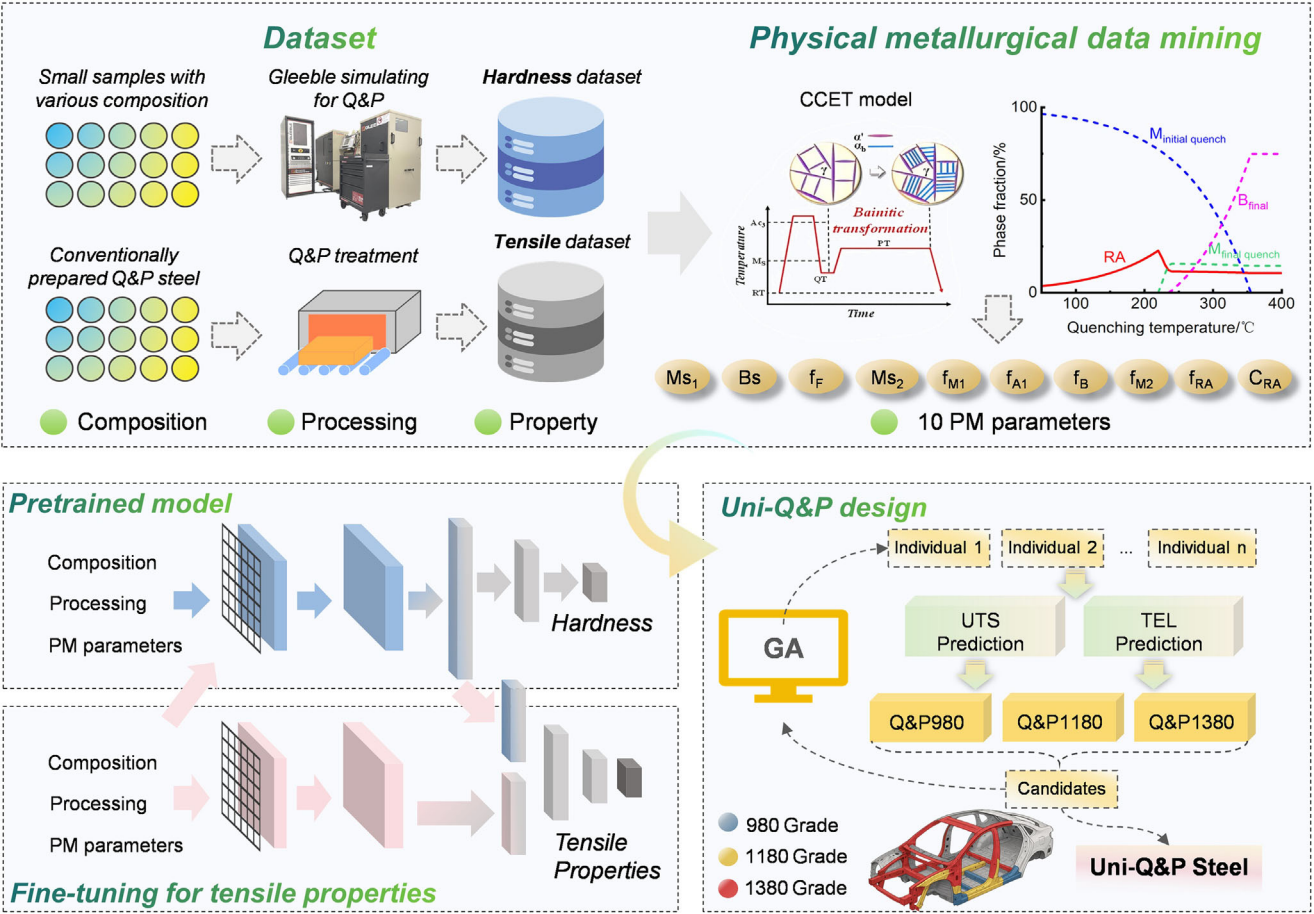
Physical metallurgy‐informed property‐bridging (PM–PB) framework for unified‐composition Q&P steel design across multiple strength grades. Rapid screening hardness and limited tensile data are coupled with CCET‐derived PM descriptors to pretrain a hardness model and transfer it to UTS/TEL prediction under sparse labels, and the resulting PB models are integrated with a multi‐objective genetic algorithm to identify one chemistry that meets Q&P980/1180/1380 targets via heat‐treatment tuning.

In this context, physics‐informed strategies that combine domain knowledge with data‐driven AI offer a promising way forward [31, 32]. In this study, a physical metallurgy‐informed AI framework is developed to enable unified‐composition Q&P steel design under data‐limited conditions (Figure 1). The approach builds on a property‐bridging (PB) concept: well‐distributed near‐high‐throughput hardness data from small Q&P samples are used to learn composition‐processing‐property relations over an extended design space, and this knowledge is then transferred to predict full‐scale tensile properties (ultimate tensile strength (UTS) and total elongation (TEL)). To ensure that the model remains anchored in metallurgical mechanisms, a set of physical metallurgy (PM) descriptors, including transformation temperatures, phase fractions, and carbon partitioning metrics, is derived from alloy composition and heat‐treatment parameters and incorporated into the learning pipeline. The resulting PM–PB framework couples physical metallurgy‐informed feature engineering with a two‐stage modeling strategy: a hardness model is first trained on the near‐high‐throughput dataset to capture broad trends in composition‐process‐property space; key representations from this model are then transferred to a tensile‐property model, effectively bridging the gap between small‐scale and large‐scale performance. This hybrid PM–PB model is subsequently embedded in a multi‐objective genetic algorithm that searches for unified Q&P steel compositions. Each candidate's chemistry is evaluated under three representative Q&P heat‐treatment schedules targeting Q&P980, Q&P1180, and Q&P1380 properties, and the optimization minimizes deviations from all three target property sets simultaneously, yielding a Pareto‐optimal family of unified‐composition designs. The top candidate is experimentally realized and shown to achieve ∼980, ∼1180, and ∼1380 MPa tensile strengths with appropriate ductility via heat‐treatment adjustments alone, thereby providing a proof‐of‐concept recyclable unified Q&P steel.

## 2 Results and Discussion

### 2.1 Mechanical Property Prediction

**FIGURE 2:**
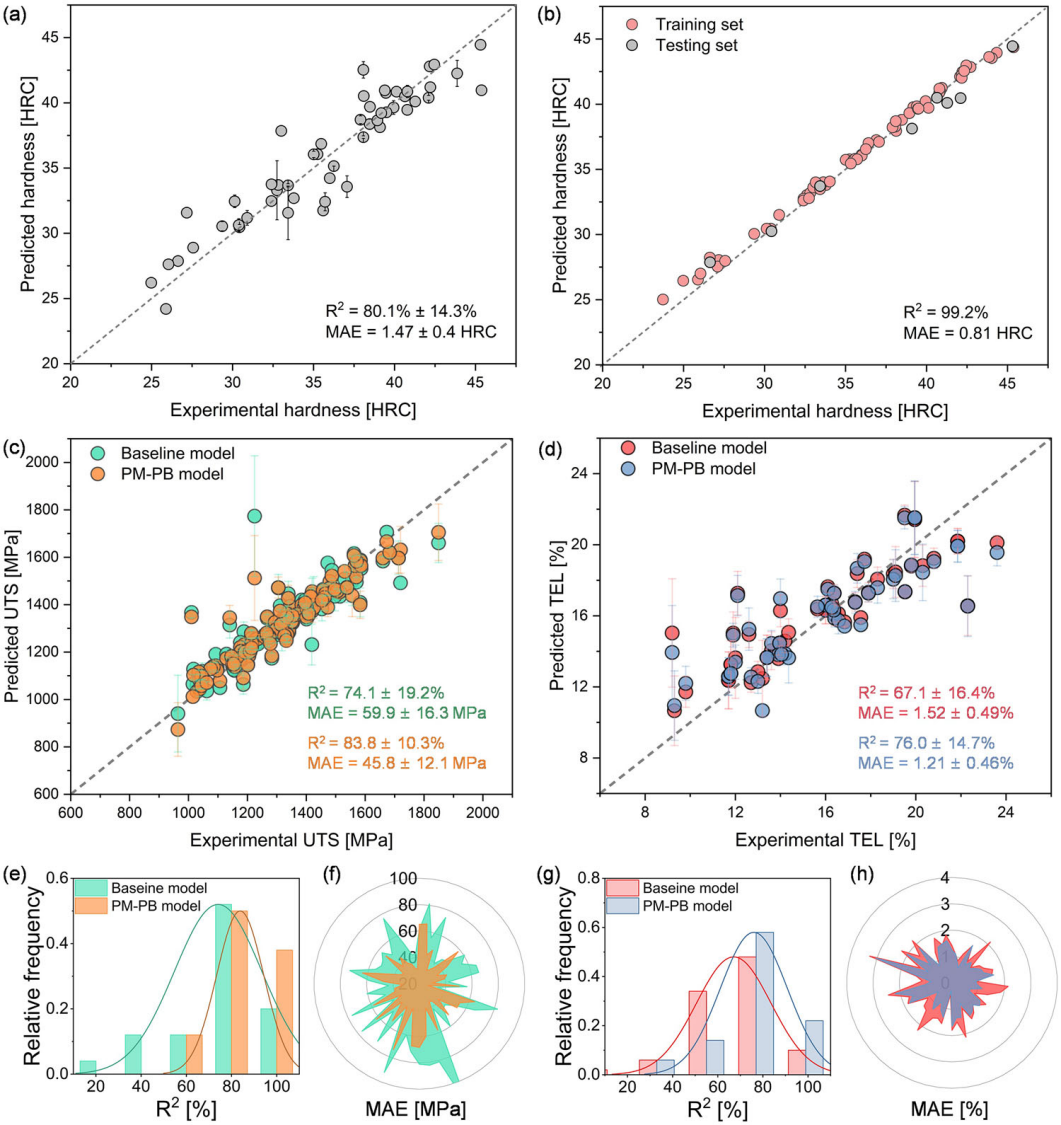
Performance of the physical metallurgy‐informed property‐bridging (PM–PB) model. (a) Averaged hardness prediction over multiple random splits. (b) hardness prediction for best‐performing model. (c) UTS prediction for testing set comparing the baseline model and the PM–PB model. (d) TEL prediction for testing set comparing the baseline and PM–PB models. (e,g) Distributions of R^2^ in testing set over 50 random splits for (e) UTS and (g) TEL. (f,h) Corresponding MAE across splits visualized in polar form for (f) UTS and (h) TEL.

Figure 2 evaluates the predictive capability and robustness of the proposed physical metallurgy‐informed property‐bridging (PM–PB) framework across hardness, tensile strength, and elongation. The workflow is first established in the hardness domain, where data are comparatively abundant and can be generated efficiently via small‐specimen testing, and is then transferred to tensile properties through the proposed PB strategy. For comparison, baseline models were constructed using only compositional and processing parameters as inputs and tensile properties as outputs, without incorporating any cross‐property knowledge transfer or physical metallurgy knowledge.

As shown in Figure 2a, the hardness model exhibits stable performance over multiple random train–test partitions, achieving an averaged R^2^ of 80.1% ± 14.3% and an MAE of 1.47 ± 0.40 HRC. The dispersion across splits mainly reflects the sensitivity of small datasets to partition choices, yet the consistently high average accuracy indicates that the model captures reproducible composition‐processing‐hardness relationships rather than overfitting to a specific split. Importantly, the best‐performing split (Figure 2b) approaches the ideal parity line with R^2^ = 99.2% and MAE = 0.81 HRC, confirming that the learned representation can faithfully describe Q&P hardenability. This well‐trained hardness model therefore provides a reliable basis for transferring knowledge to tensile property prediction, where labels are typically scarcer and more expensive to obtain.

Building on the hardness‐pretrained representation, the PB strategy substantially improves tensile strength prediction relative to a purely data‐driven baseline. In Figure 2c, the baseline model shows pronounced scatter and weaker generalization to unseen samples, with R^2^ = 74.1% ± 19.2% and MAE = 59.9 ± 16.3 MPa. By contrast, the PM‐PB model delivers higher and more stable accuracy (R^2^ = 83.8% ± 10.3%) while reducing the prediction error (MAE = 45.8 ± 12.1 MPa). Notably, the reduction in prediction dispersion becomes increasingly significant as the data support per compositional system decreases (In other words, the data becomes increasingly sparse), as shown in Figure S1. This trend directly validates the central hypothesis of the PM–PB framework: pretraining in a densely sampled hardness domain, together with the incorporation of physical metallurgy features, introduces a physically meaningful inductive bias that enables the model to generalize beyond the limited tensile training distribution and remain robust under data‐scarce conditions.

A similar, and practically significant, improvement is observed for elongation. As shown in Figure 2d, the baseline model exhibits larger dispersion and limited reliability for TEL, consistent with the well‐known sensitivity of ductility to subtle microstructural variations in Q&P steels. Incorporating PB transfer learning and physical metallurgy descriptors yields a clear gain in predictive performance, with reduced error relative to the baseline. The improvement is mechanistically plausible: TEL in Q&P steels depends strongly on transformation‐assisted plasticity and microstructural stability. Embedding transformation‐relevant descriptors within the PM–PB framework therefore, provides an effective route to learn ductility trends that are otherwise difficult to infer from sparse tensile labels alone.

Figure 2e–h further substantiate the robustness advantage of PM–PB under repeated random splits. The distributions of testing R^2^ over 50 splits (Figure 2e,g) shift toward higher values for PM‐PB compared with the baseline, indicating improved reliability rather than isolated best‐case gains. Consistently, the corresponding MAE statistics visualized in polar form (Figure 2f,h) reveal reduced error magnitudes under almost all splits and fewer high‐error “spikes” for PM–PB. Taken together, these distribution‐level evidences demonstrate that the proposed framework improves not only average accuracy but also stability with respect to data partitioning, which is essential for model‐driven alloy design where decisions depend on trustworthy predictions across diverse processing conditions.

To further examine the robustness of the PM–PB model, two additional validation protocols were considered: a composition‐family‐based split and a nested cross‐validation analysis. Although the absolute R^2^ values decreased under the stricter composition‐family‐based split, the PM‐PB model still outperformed the purely data‐driven baseline for UTS prediction (Figure S2). Consistently, the superiority of PM‐PB was also preserved under nested cross‐validation. These results suggest that random splitting may overestimate out‐of‐family generalization, while the advantage of the PM–PB framework remains robust across different validation protocols. Additional source‐heterogeneity analysis further showed that, although inter‐source variability is present in the dataset, the deviations are not uniformly directional across sources, and the relative advantage of PM–PB over the baseline remains observable for a substantial fraction of data sources (Figure S3).

To gain mechanistic insight into the physics‐guided nature of the PM–PB framework, SHAP analysis was performed on the final UTS and TEL prediction models. The most influential features include several physical‐metallurgy descriptors, particularly Ms_1_, Bs, *f*_F_ and *f*_B_, together with key processing and compositional variables (Figure S4). Notably, PM descriptors account for most of the top‐ranked features, indicating that the model relies strongly on descriptors associated with transformation temperatures, phase evolution, and retained‐austenite stabilization rather than only on nominal composition or heat‐treatment parameters. This result provides direct interpretive support for the physics‐guided character of the PM–PB framework. It should be noted, however, that these PM descriptors are not direct measurements of the evolving microstructural state, but approximate physics‐informed features derived from thermodynamic calculations and empirical transformation relations. Therefore, their value in the present framework lies primarily in encoding physically meaningful trends and constraints for data‐sparse learning, rather than in exact reconstruction of the underlying non‐equilibrium microstructure.

Overall, Figure 2 shows that PM‐PB provides a robust and accurate predictive framework for hardness, UTS, and TEL in Q&P steels by coupling physics‐guided descriptors with property‐bridging transfer learning. This performance foundation supports the subsequent multi‐objective optimization of unified‐composition Q&P steels, where the search efficiency and the credibility of the proposed candidates depend on stable tensile‐property predictions across the explored process space. Given the limited dataset size, however, these results should be interpreted as demonstrating the feasibility and value of the PM‐PB framework within the explored Q&P steel composition–process space, rather than unrestricted generalizability to arbitrary alloy systems.

### 2.2 Design and Experimental Validation of Unified Q&P Steel

**FIGURE 3:**
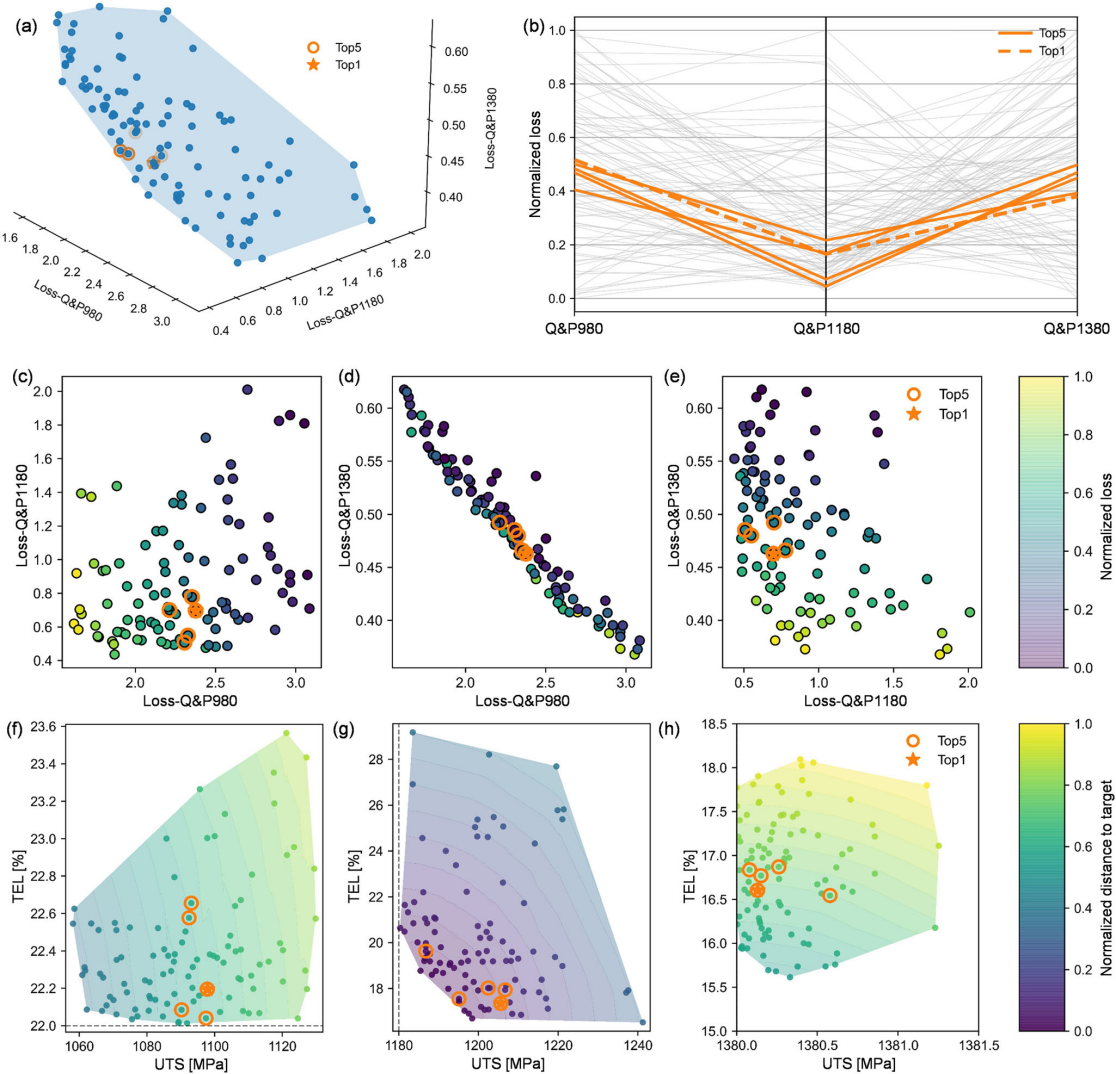
Multi‐objective optimization and property‐space evaluation of unified Q&P steel designs based on the ideal‐point criterion. (a) Three‐dimensional Pareto front in the loss space of Q&P980, Q&P1180, and Q&P1380. The shaded surface represents the non‐dominated front. Circles denote the Top‐5 solutions and the star indicates the Top‐1 solution selected by minimizing the Euclidean distance to the ideal point (zero loss for all grades). (b) Parallel‐coordinates plot of normalized grade‐specific losses. Gray lines represent all candidate solutions, while orange lines highlight the Top‐5 ideal‐point solutions, with the dashed line indicating the Top‐1 design. The selected solutions exhibit balanced loss profiles across the three grades. (c–e) Pairwise projections of the Pareto front colored by the normalized value of the third loss objective, illustrating trade‐offs among grade‐specific performances. Ideal‐point solutions are located near the central knee‐like region of the Pareto front. (f–h) UTS–TEL property spaces for Q&P980, Q&P1180, and Q&P1380. The color scale represents the normalized distance to the target performance, defined as the Euclidean distance to the target point in the UTS‐TEL plane after normalization by the respective ranges of UTS and TEL (smaller values indicate closer agreement). The ideal‐point solutions consistently approach the target regions across all three grades.

To realize a unified Q&P steel chemistry that can be tuned to multiple strength grades (Q&P980/Q&P1180/Q&P1380) through heat‐treatment adjustment, a tri‐objective optimization problem was formulated, in which the objective vector consists of the prediction losses for the three target grades, with all objectives minimized simultaneously. Candidate solutions were generated using a genetic algorithm and evaluated by the trained performance models, producing a population distributed in the three‐dimensional objective space (Figure 3a). Details of the genetic algorithm design are provided in the Methods section. The resulting distribution exhibits a clear trade‐off surface, confirming the intrinsic multi‐regime coupling between composition, processing, and mechanical response in Q&P steels.

Figure 3a presents the non‐dominated set (Pareto front) in the loss space. Rather than filling the entire objective volume, feasible solutions concentrate on a relatively thin manifold, indicating that the model‐guided search rapidly converges toward metallurgically plausible trade‐offs constrained by shared underlying physical mechanisms rather than arbitrary numerical correlations.

Beyond geometric proximity in the objective space, the balance among objectives is more explicitly revealed by the parallel‐coordinates plot (Figure 3b), where the three normalized grade‐level losses are displayed simultaneously. Here, an ideal‐point criterion is adopted to identify representative unified designs by minimizing the Euclidean distance to the ideal point, defined as zero loss for all three grades. This criterion favors solutions that achieve simultaneously low losses across Q&P980, Q&P1180, and Q&P1380, rather than solutions that are strongly biased toward a single grade. Accordingly, the Top‐5 ideal‐point solutions are highlighted as balanced candidates, with the Top‐1 solution corresponding to the minimum overall distance to the ideal point. Compared with the majority of candidate solutions that exhibit tilted or dispersed profiles, the ideal‐point–selected solutions display compact and relatively flat loss profiles, directly visualizing their balanced multi‐grade performance.

The pairwise projections of the Pareto front (Figure 3c–e) further elucidate the structure of the trade‐offs. The point clouds are elongated along specific directions rather than isotropically distributed, revealing structured antagonism between objectives. This anisotropic geometry suggests that the optimization is governed by a limited number of latent metallurgical degrees of freedom–‐such as the balance between hardenability and retained‐austenite stability–‐that influence different grades with unequal sensitivities. Notably, the ideal‐point solutions cluster in regions where further improvement in one objective would require increasingly disproportionate sacrifices in others, indicating a physically meaningful compromise among competing performance requirements.

While the Pareto plots quantify trade‐offs in the loss space, practical alloy selection must ultimately be interpreted in the property space under grade‐specific strength–ductility targets. Figure 3f–h visualize the candidate solutions in the UTS–EL space for Q&P980, Q&P1180, and Q&P1380, respectively, with target intersections indicated by dashed reference lines. The color scale represents the normalized distance to the target point in each UTS–EL plane. The ideal‐point–selected solutions consistently lie close to the target regions for all three grades, demonstrating that the selected chemistry can be tuned through heat‐treatment adjustment while maintaining balanced proximity to the desired strength–ductility combinations across grades.

Based on this multi‐objective screening and property‐space consistency check, a unified alloy chemistry was identified (Table 1). Rather than varying chemistry across grades, different strength levels are achieved by adjusting the Q&P schedule (AT/QT/PT/Pt), resulting in three representative processing windows. This strategy explicitly separates the roles of chemistry and processing: the chemistry provides a common metallurgical backbone in terms of hardenability and retained‐austenite stabilization capability, while processing modulates martensite fraction, carbon partitioning, and retained‐austenite stability to reach different strength–ductility combinations. Collectively, the Pareto‐based selection, ideal‐point balance criterion, and grade‐specific property‐space validation provide quantitative evidence that a single chemistry can support a multi‐grade Q&P portfolio through controlled heat‐treatment modulation.

**TABLE 1:** Composition and Q&P treatment processing parameters of newly designed alloy.

C (wt.%)	Mn (wt.%)	Si (wt.%)	Cr (wt.%)	Mo (wt.%)	Nb (wt.%)	AT (°C)	QT (°C)	PT (°C)	Pt (s])
0.23	1.98	1.5	0.2	0	0.01	785	284	365	180
						828	210	479	180
						920	247	369	180

For experimental validation, the top‐ranked unified composition (Top‐1) was fabricated and subjected to three designed Q&P heat‐treatment schedules (Table 1) corresponding to the 980, 1180, and 1380 MPa grade targets.

**FIGURE 4:**
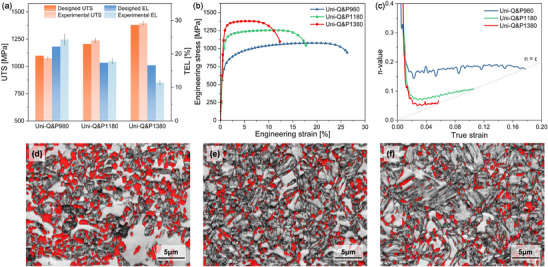
Experimental validation of the unified Q&P steel across multiple strength grades. (a) Comparison between experimentally measured and model‐predicted UTS and TEL for Q&P980, Q&P1180, and Q&P1380. (b) Representative stress–true curves of the three strength grades, demonstrating the tunable strength–ductility balance achieved through heat‐treatment adjustment. (c) Evolution of the work‐hardening exponent (*n*‐value) as a function of true strain, revealing grade‐dependent strain‐hardening behavior. (d–f) EBSD phase maps of Q&P980, Q&P1180, and Q&P1380, respectively, where retained austenite is highlighted in red. The corresponding retained‐austenite fractions measured by XRD are 15.2%, 10.7%, and 10.8% for Q&P980, Q&P1180, and Q&P1380, respectively.

The measured UTS and TEL values show close agreement with the PM‐PB model's predictions (Figure 4a), with all data points falling within normal experimental scatter. More importantly, the model correctly captures the systematic evolution of the strength–ductility balance with increasing strength level, confirming that the learned composition–processing–property relationships remain transferable across different Q&P processing windows rather than being confined to a single grade. The engineering stress–strain curves in Figure 4b further reveal grade‐dependent deformation characteristics governed by processing. Q&P980 exhibits an extended uniform deformation regime with gradual strain hardening, whereas Q&P1180 shows a balanced combination of elevated strength and sustained plasticity. In contrast, Q&P1380 displays a high initial flow stress accompanied by a reduced but stable uniform elongation. The *n*‐value evolution (Figure 4c) exhibits a characteristic non‐monotonic trend, with an initial decrease followed by partial recovery or stabilization at intermediate strains, reflecting the delayed activation of TRIP‐assisted hardening. The reduced recovery observed for Q&P1380 indicates a progressively constrained TRIP contribution with increasing strength level.

EBSD phase maps (Figure 4d–f) reveal a finely dispersed retained austenite (RA) phase embedded within the martensitic matrix for all three grades. XRD measurements indicate RA fractions of 15.2%, 10.7%, and 10.8% for Q&P980, Q&P1180, and Q&P1380, respectively. The systematic reduction in RA fraction with increasing strength level is consistent with the observed decrease in uniform elongation and work‐hardening capacity. Notably, even at the highest strength level, RA remains spatially well distributed rather than forming coarse isolated regions, enabling residual TRIP contributions and stabilizing plastic deformation.

Overall, the combined prediction‐experiment consistency, grade‐dependent stress‐strain and work‐hardening behavior, and microstructural evidence collectively demonstrate that a single alloy chemistry can support multiple strength grades through controlled Q&P heat‐treatment modulation. This unified design strategy decouples chemistry from strength level, allowing processing to tune martensite fraction, carbon partitioning, and RA stability, thereby enabling a scalable multi‐grade Q&P steel portfolio.

### 2.3 Ablation Study and Data‐Efficiency Analysis

**FIGURE 5:**
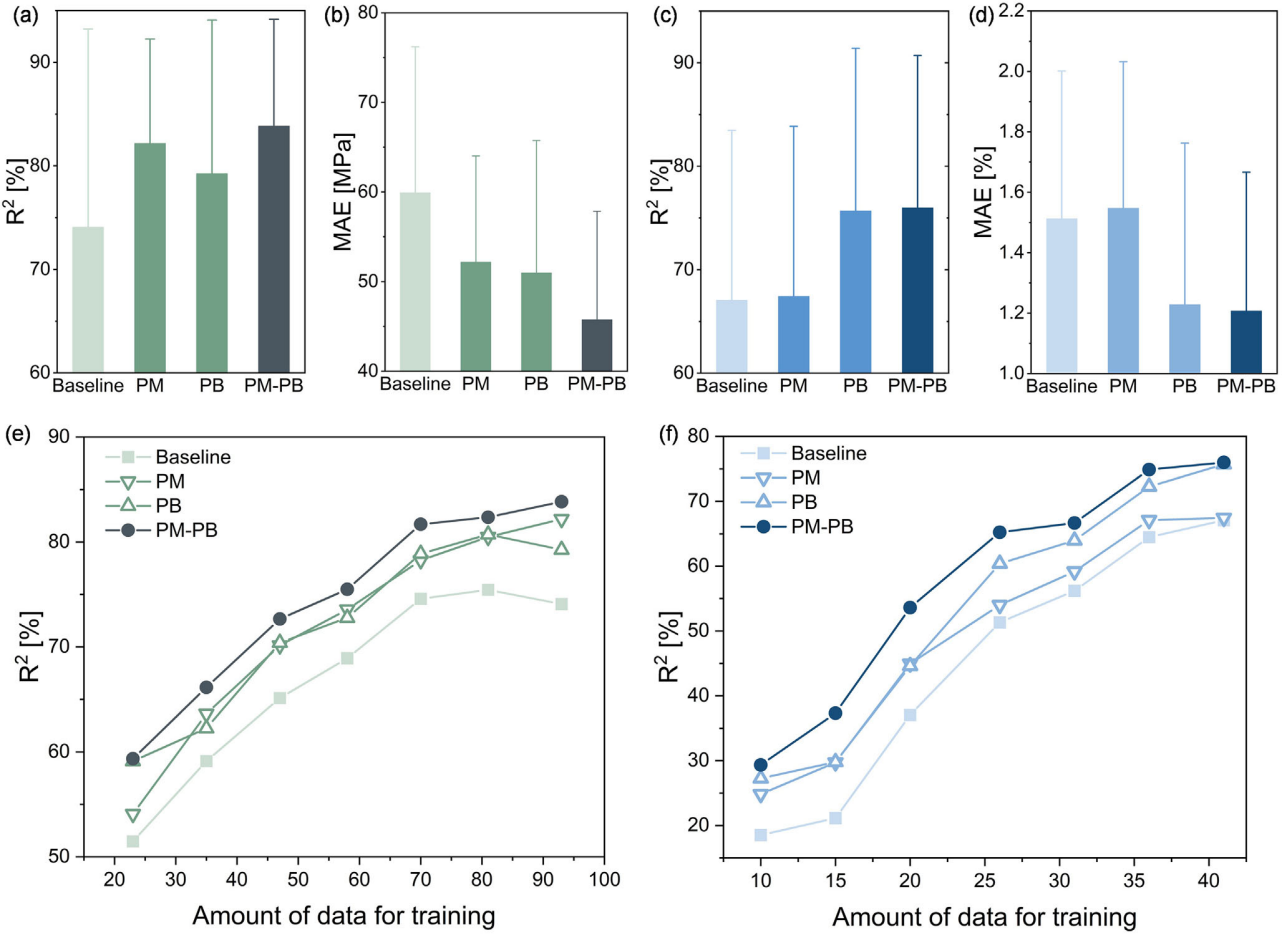
Performance comparison and data‐scaling behavior of PM‐PB models. (a) Testing R^2^ and (b) MAE for UTS predicted by the baseline, PM, PB, and PM‐PB models, averaged over multiple random splits. Corresponding testing (c) R^2^ and (d) MAE for TE. Dependence of testing R^2^ on the amount of training data for (e) UTS and (f) TEL.

To elucidate why the proposed PM‐PB framework enables reliable multi‐grade design under limited data conditions, an ablation study and data‐efficiency analysis were conducted. Four model configurations were evaluated: a baseline purely data‐driven model, a PM‐only model incorporating transformation‐related descriptors, a PB‐only model leveraging hardness pretraining, and the full PM‐PB model combining both enhancements. Figure 5a,b compares the predictive performance for UTS across four model variants. The baseline model, relying solely on compositional and processing inputs, exhibits limited accuracy and large uncertainty. Incorporating PM descriptors leads to a systematic improvement, indicating that embedding transformation temperatures, phase fractions, and carbon‐partitioning–related information introduces physically meaningful inductive bias into the learning process. A larger performance gain is achieved by the PB‐only model, confirming that hardness‐based pretraining provides a more informative representation of the composition–processing space. The full PM–PB model delivers the highest R^2^ and lowest MAE, with noticeably reduced variance, demonstrating that mechanistic descriptors and cross‐property transfer act synergistically rather than redundantly.

An analogous trend is observed for TEL prediction (Figure 5c,d), although the overall task is more challenging due to the strong sensitivity of ductility to microstructural heterogeneity in Q&P steels. The baseline model shows pronounced scatter, reflecting the difficulty of capturing elongation from limited tensile data alone. The PM‐only model slightly improves stability by explicitly encoding retained‐austenite stability and transformation‐induced plasticity effects, while the PB‐only model further enhances accuracy and plays a major role by transferring information implicitly related to martensite fraction and work‐hardening behavior. The combined PM‐PB model consistently achieves the most accurate and robust TEL predictions, capturing both low‐ and high‐ductility regimes with reduced bias.

The data‐efficiency analysis in Figure 5e,f provides further insight into the origin of this performance advantage. When the training dataset is progressively reduced, the baseline model rapidly deteriorates, highlighting its reliance on purely statistical correlations. Introducing PM descriptors slows this degradation by constraining the hypothesis space to metallurgically plausible relationships. The PB‐only model exhibits strong early‐stage performance, indicating that hardness‐based pretraining supplies an effective initialization that mitigates overfitting. Notably, the full PM‐PB model maintains the highest R^2^ values across all training set sizes and remains reliable even with severely limited data, directly supporting the design scenarios explored in Figure 3.

Taken together, Figure 5 rationalizes the effectiveness of the PM‐PB framework. The PM descriptors encode essential metallurgical mechanisms that govern microstructure evolution and deformation behavior, while the PB strategy leverages near‐high‐throughput hardness data to overcome the intrinsic scarcity of tensile measurements. Their integration yields a data‐efficient and physically grounded predictor, providing a robust foundation for multi‐objective optimization and unified Q&P steel design. This advantage is not restricted to repeated random train/test splits. Additional validation using a composition‐family‐based split and nested cross‐validation also preserved the superiority of PM‐PB over the baseline. These results suggest that the benefit of combining physical metallurgy descriptors with property‐bridging transfer learning is not merely an artifact of a favorable splitting strategy.

## 3 Conclusion

This study establishes a physics‐informed and data‐efficient framework for unified‐composition Q&P steel design, enabling multiple strength grades to be realized from a single alloy chemistry through heat‐treatment tuning alone. By combining CCET‐derived physical‐metallurgy descriptors with a property‐bridging transfer strategy from near‐high‐throughput hardness data, the PM‐PB model delivers accurate and robust tensile predictions under sparse tensile labels, providing a reliable surrogate for multi‐objective optimization. A tri‐objective genetic algorithm guided by the PM‐PB model identifies an ideal‐point unified chemistry that experimentally achieves Q&P980, Q&P1180, and Q&P1380 property targets via tailored Q&P schedules. Mechanical testing and microstructural characterization confirm grade‐dependent strength‐ductility responses governed by controlled martensite fraction and retained‐austenite stability, validating the transferability of the learned composition–process–property relationships. This work demonstrates a general design principle for sustainable alloys: embedding physical metallurgy into cross‐property machine learning enables credible multi‐grade design while reducing chemistry proliferation, offering a scalable pathway toward recycling‐friendly structural materials.

## 4 Methods

### 4.1 Materials and Dataset Construction

The development of the proposed PM‐PB framework relies on two complementary datasets that together capture both rapid screening small‐sample hardness and full‐scale tensile properties of Q&P steels.

To construct a near‐high‐throughput dataset that captures the coupled composition‐processing‐hardness response of Q&P steels, small cylindrical specimens were designed to systematically span a low‐alloy chemistry space and a practically relevant Q&P processing window. All alloys were prepared with controlled compositions, homogenized, and subsequently subjected to quenching and partitioning (Q&P) cycles using a Gleeble thermomechanical simulator, which provides high‐precision thermal control and repeatable heat‐treatment schedules. The Gleeble program independently varied the austenitization temperature (AT), quench temperature (QT), and partitioning temperature/time (PT/Pt), thereby enabling broad coverage of industrially meaningful Q&P schedules. The dataset inputs consist of (i) alloy chemistry in wt.% (C, Mn, Si, Al, Cr, Mo, and Nb) and (ii) Q&P processing parameters (AT, QT, PT, and Pt). The output is the post‐treatment Rockwell hardness measured in the HRC scale. In total, 72 unique composition‐processing conditions were generated. The investigated composition ranges are summarized in Table S1, where C (0.10‐0.30 wt.%), Mn (1.80‐3.20 wt.%), Si (1.00‐2.00 wt.%), and Al (0.04‐0.80 wt.%) act as the primary design variables. Processing parameters were varied over AT = 760–900°C, QT = 180–230°C, and PT = 350–450°C, with Pt fixed at 100 s. The resulting dataset spans a wide hardness range from 23.7 to 45.4 HRC, reflecting substantial variations in Q&P microstructural states across the explored design space. Figure S5 further visualizes the pairwise distributions among composition, processing parameters, and hardness, confirming that the 72 conditions provide broad and well‐dispersed coverage of the explored composition‐processing space.

In parallel, two tensile‐property sub datasets were compiled for ultimate tensile strength (UTS) and total elongation (TEL). The data sources include: (i) a curated collection of high‐quality literature reports on Q&P heat‐treatment routes, and (ii) tensile data accumulated from our prior experimental work. Tensile tests were conducted using A50 specimen geometry under ASTM E8 standard (or equivalent reporting in the literature), enabling meaningful comparison of tensile properties across studies. For each tensile record, the input features are the same as those in the hardness dataset. The final UTS dataset contains 117 composition‐processing‐property records, while the TEL dataset contains 52 records. Tables S2 and S3 summarize the statistical ranges of all input and output variables, indicating that the compiled datasets span broad chemistry and processing windows as well as wide property variations representative of multi‐grade Q&P steels. Figures S6 and S7 further visualize the pairwise distributions and correlations between inputs and tensile outputs, confirming a well‐dispersed coverage of the composition‐processing space.

### 4.2 Physical‐Metallurgical Descriptor Extraction

To embed metallurgical interpretability into the property‐bridging models, a set of physics‐based PM descriptors was extracted for each alloy‐processing condition to represent the key transformation pathway in Q&P steels. Specifically, ten PM descriptors were calculated, including the primary and secondary martensite start temperatures (Ms_1_ and Ms_2_), bainite start temperature (Bs), phase fractions associated with incomplete austenitization and subsequent transformations (*f*_F_, *f*_M1_, *f*_A1_, *f*_B_, *f*_M2_, and *f*_RA_), and the carbon concentration in retained austenite (*C*_RA_). These descriptors collectively capture the sequence of austenitization, quenching‐induced martensite formation, potential bainitic transformation during partitioning, carbon redistribution and austenite stabilization, and secondary martensite formation upon final cooling.

The PM descriptor calculation integrates thermodynamic equilibrium calculations at AT (Thermo‐Calc with TCFE9 database), empirical transformation relations for Ms and Bs [39, 40], and a constrained‐carbon‐equilibrium framework with the *T*_0_ criterion (CCET model [41]) to account for carbon partitioning limits during the partitioning step. In brief, equilibrium phase fractions at AT were first evaluated to correct the effective carbon content dissolved in austenite, which was then used to compute Ms_1_ and Bs and to estimate the primary martensite fraction *f*_M1_ formed during quenching. Partitioning was subsequently modeled by enforcing carbon redistribution under the CCET constraint, with bainitic competition considered when thermodynamically feasible, yielding *C*_RA_ and the remaining austenite fraction *f*_A1_ after partitioning. Finally, Ms_2_ and the secondary martensite fraction *f*_M2_ during cooling were evaluated, and the final retained austenite fraction *f*_RA_ was obtained by phase‐fraction closure. Full equations, assumptions, and parameter settings are provided in the Supporting Information.

To validate the physical consistency and added value of the extracted PM descriptors, their distributions and feature‐property relations were systematically examined. Tables S1–S3 summarize the statistical ranges of PM descriptors for the hardness, UTS, and TEL datasets, while the pairwise correlation maps (Figures S8–S10) confirm broad and well‐dispersed coverage of the metallurgical descriptor space. Representative alloys processed under identical Q&P conditions in the hardness dataset further show physically consistent trends, where higher retained‐austenite fraction and carbon enrichment (*C*_RA_≈1.38–1.96 wt.%) generally correspond to higher hardness, whereas higher ferrite fraction leads to reduced hardness (Figure S11). In addition, Pearson correlations and random forest‐based mean decrease accuracy (MDA) rankings (Figures S12–S15) reveal coherent and metallurgy‐aligned dependencies between both composition‐processing variables and PM descriptors with hardness/strength/ductility, with PM features emerging as dominant and complementary predictors once introduced. Collectively, these analyses demonstrate that the PM descriptors encode mechanistically meaningful, non‐redundant transformation information beyond raw composition and processing parameters, thereby supporting their use as physically interpretable inputs for PB modeling. At the same time, these descriptors remain approximate because they rely on simplified thermodynamic/empirical relations and limited assumptions regarding non‐equilibrium transformation behavior during Q&P processing. Accordingly, they should be interpreted as physically informed surrogate features rather than exact state variables, and their uncertainty may propagate into the downstream predictive models.

### 4.3 Property‐Bridging Modeling Strategy

The proposed PB framework consists of two stages: (i) pretraining a source convolutional neural network (Source‐CNN) on the hardness dataset to learn a transferable representation of composition‐processing‐PM descriptors; and (ii) constructing a PB transfer network (PB‐CNN) for tensile‐property prediction (UTS and TEL) by reusing the pretrained source representation and learning a task‐specific correction branch with sparse tensile labels. For each alloy‐processing condition, the input vector (21 features: composition, Q&P processing parameters, and PM descriptors) was standardized (z‐score) and then zero‐padded and reshaped into a 5×5 feature map to enable convolutional feature extraction. Based on random‐forest screening (Figure S16), the full set of 10 PM descriptors was adopted for PB modeling, as performance gains saturated without degradation upon feature addition.

The Source‐CNN was trained using the mean absolute error (MAE) loss and the Adam optimizer, and the best model was selected by minimizing the validation loss. For tensile‐property modeling, the PB‐CNN integrates (a) a frozen source encoder that outputs the intermediate embedding from the pretrained Source‐CNN and (b) a trainable target encoder that learns complementary task‐specific features from the same standardized inputs. The two embeddings are concatenated and mapped to the tensile output through lightweight fully connected layers.

For the hardness dataset, the samples were divided into training and testing sets in a 9:1 ratio. The process was repeated ten times randomly, and the optimal model was selected to establish the PB model. For the UTS and TEL datasets, the samples were then divided into training and testing sets in an 8:2 ratio. This splitting process was repeated randomly 50 times. Model performance was evaluated using two key metrics: the squared correlation coefficient (R^2^) and MAE, which were calculated as follows:

(1) $$\begin{array}{c} R^{2}=\frac{{\left(n\sum_{i=1}^{n}f\left(x_{i}\right)y_{i}-\sum_{i=1}^{n}f\left(x_{i}\right)\sum_{i=1}^{n}y_{i}\right)}^{2}}{\left(n\sum_{i=1}^{n}f{\left(x_{i}\right)}^{2}-{\left(\sum_{i=1}^{n}f\left(x_{i}\right)\right)}^{2}\right)\left(n\sum_{i=1}^{n}{y_{i}}^{2}-{\left(\sum_{i=1}^{n}y_{i}\right)}^{2}\right)} \end{array}$$

(2) $$MAE=\frac{1}{n}\sum_{i=1}^{n}\left|f\left(x_{i}\right)-y_{i}\right|$$

For comparison, a purely data‐driven CNN baseline was constructed for tensile‐property prediction. This model was trained directly on the tensile‐property datasets without PB or PM. In addition to this original baseline, several conventional machine‐learning models were further introduced as benchmark methods, including XGBoost (XGB), Random Forest (RF), Gradient Boosting Regression (GBR), Support Vector Regression (SVR), and Fully Connected Neural Network (FCNN). These benchmark models were selected because they represent a diverse set of widely used regression algorithms for small‐to‐medium‐sized materials datasets, including tree‐based, kernel‐based, and neural‐network‐based approaches. These models were trained for both UTS and TEL prediction using the same input settings as the baseline model. The hyperparameters were optimized by cross‐validation. To ensure fair comparison, all models were evaluated using the same repeated random‐split protocol as that used for the tensile‐property prediction task. The comparative results for additional benchmark models are summarized in Figure S17.

Full architectural specifications of the Source‐CNN, PB‐CNN, and CNN baseline are provided in Tables S4–S6, respectively, together with the associated training hyperparameters and reproducibility settings in the Supporting Information.

To further evaluate potential source‐dependent bias in the dataset, each data point was assigned a source label according to its origin, and residuals were analyzed on a source‐by‐source basis. Source‐wise mean residuals and MAE were compared between the purely data‐driven baseline model and the PM‐PB model, and the source‐wise MAE improvement of PM‐PB over the baseline was further examined.

### 4.4 Unified Q&P Steel Design and Experimental Validation

A unified‐composition design was achieved by coupling the physical metallurgy‐informed PM–PB model with a multi‐objective genetic algorithm (GA). For each candidate alloy chemistry, the PB model predicts UTS and TEL under three representative Q&P heat‐treatment schedules targeting the Q&P980, Q&P1180, and Q&P1380 grades. The GA evaluates each candidate by the deviation of the predicted properties from the corresponding target windows (Q&P980: 980 MPa and 22% for UTS and TEL; Q&P1180: 1180 MPa and 15.5% for UTS and TEL; Q&P1380: 1380 MPa and 12% for UTS and TEL), treating the selected grade‐level criteria as simultaneous objectives.

For each grade, the loss function of GA was defined as a composite metric combining strength and elongation deviations,

(3) $$L_{i}=\frac{\left|\Delta UTS_{i}\right|}{50}+\frac{\left|\Delta TEL_{i}\right|}{10}$$

where Δ*UTS_i_* and Δ*TEL_i_* denote the differences between predicted and target ultimate tensile strength and elongation for grade *i*, respectively. This formulation normalizes the relative contributions of strength and ductility and reflects their comparable importance in Q&P steel design.

Alloying elements were encoded as continuous design variables constrained within industry‐feasible bounds. The population was evolved via selection, crossover, and mutation, with Pareto dominance and crowding‐distance sorting used to preserve both optimality and diversity. The optimization yields a Pareto set of unified‐composition steels that can meet multiple strength‐ductility targets through schedule variation. A top‐ranked composition with balanced performance was selected for experimental validation.

The optimal alloy was melted in a vacuum induction furnace (50 kg ingots), forged at 1100°C into billets (200 × 135 mm^2^), reheated at 1200°C for 4 h, and hot‐rolled to 4 mm thickness, followed by furnace cooling to 550°C. The plate was then cold‐rolled to 1.5 mm thickness. Q&P heat treatments (austenitization, quenching, and partitioning) were then conducted according to the GA‐derived schedules. Tensile tests were performed using ASTM E8 subsize A50 specimens.

Microstructures were characterized by scanning electron microscopy (SEM) and electron backscatter diffraction (EBSD). For SEM, samples were mechanically polished and etched in 4% nital, and examined using a JEOL JSM‐7200F microscope. For EBSD, samples were prepared by electropolishing in perchloric acid‐ethanol. Retained austenite (RA) fractions were quantified by X‐ray diffraction (XRD). The RA volume fraction was calculated using the equations reported in present works [42, 43, 44].

## Conflicts of Interest

The authors declare no conflicts of interest.

## Supporting information




**Supporting File**: advs75515‐sup‐0001‐SuppMat.docx.

## Data Availability

The data that support the findings of this study are available from the corresponding author upon reasonable request.
