# Supplementary material for: Physics‐Informed Machine Learning for Sustainable Alloy Design: Toward a Recyclable Unified Q&P Steel
Source: Adv Sci (Weinh). 2026 Jun 15:e75515. Online ahead of print. doi: 10.1002/advs.75515 (PMC13335544; doi:10.1002/advs.75515)
Supplement: Supplementary file 1 — Supporting File: advs75515‐sup‐0001‐SuppMat.docx. [file ADVS-9999-e75515-s001.docx]

Supporting Information

Physics-Informed Machine Learning for Sustainable Alloy Design: Toward a Recyclable Unified Q&P Steel

Xiaolu Wei, Yong Li, Chenchong Wang*, Lingyu Wang^1^, Xiang Song, Keming Mao, Yu Zhang and Wei Xu*

X. Wei, Y. Li, C. Wang, L. Wang, X. Song, W. Xu

The State Key Laboratory of Digital Steel, Northeastern University, Shenyang, China
E-mail: wangchenchong@ral.neu.edu.cn; xuwei@ral.neu.edu.cn

K. Mao

Software College, Northeastern University, Shenyang, China

Y. Zhang

Ansteel Beijing Research Institute Co., Ltd., Beijing, China

1. **Dependence of prediction on data sparsity across compositional systems**


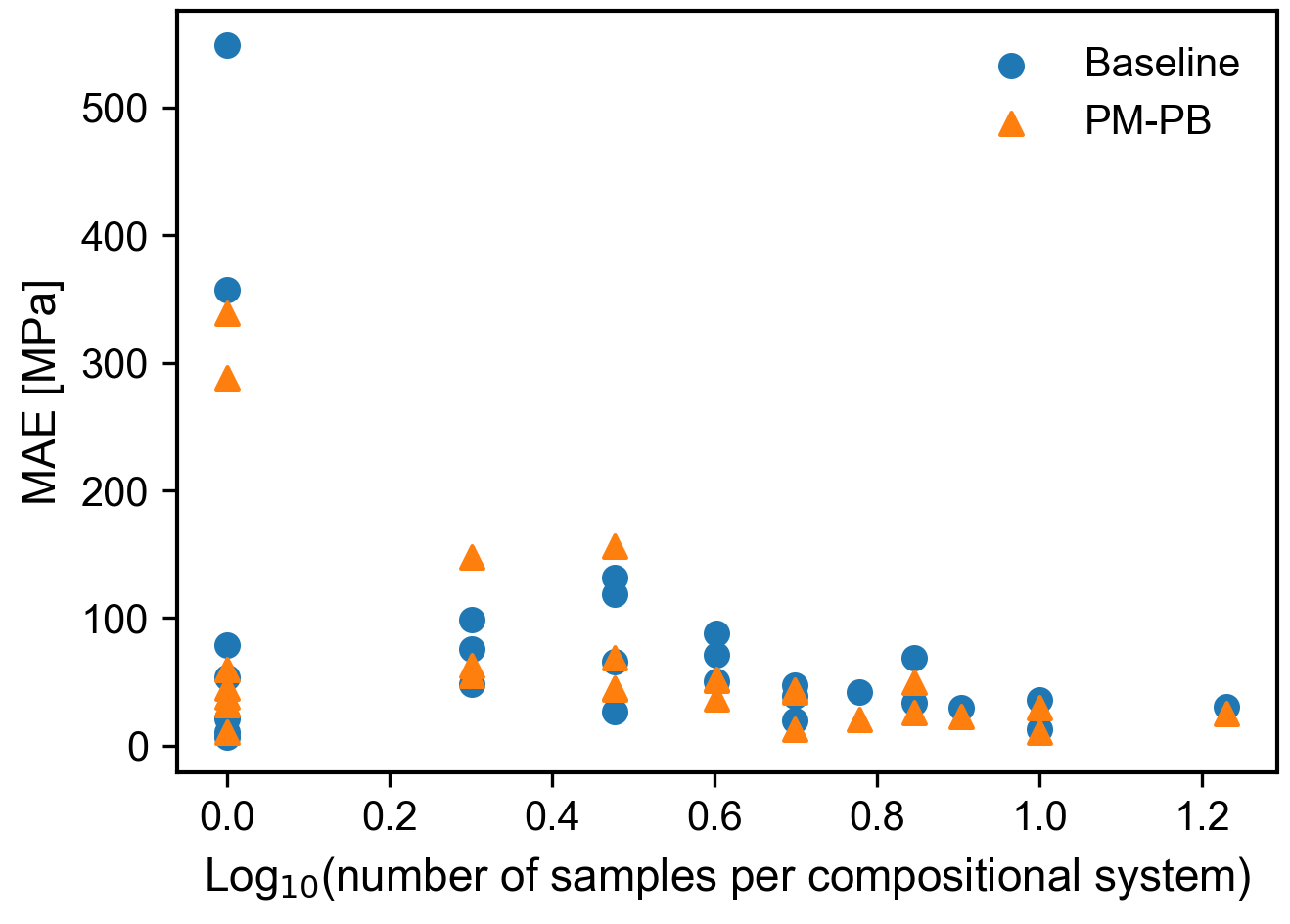


Figure S1. Dependence of prediction error on data sparsity across compositional systems.

1. **Additional validation under different data partitioning protocols**

To examine the sensitivity of the reported UTS performance to the choice of data partitioning strategy, two additional validation protocols were considered beyond the repeated random train/test splits used in the main text: (i) a composition-family-based split and (ii) a nested cross-validation analysis.

In the composition-family-based split, samples with the same nominal alloy chemistry (C, Mn, Si, Al, Cr, Mo, and Nb) were assigned to the same group and were not allowed to appear simultaneously in the training and test sets. This protocol provides a stricter estimate of out-of-family generalization by reducing compositional leakage between the training and testing data. In the nested cross-validation analysis, an outer shuffled cross-validation loop was used for model evaluation, while a small inner validation split within each outer training fold was used for limited model selection among candidate hyperparameter configurations.

Figure S2 summarizes the UTS prediction performance of the baseline and PM-PB models under the three validation protocols. Compared with repeated random splitting, the composition-family-based split led to substantially lower absolute R^2^ values for both models, indicating that conventional random splitting can overestimate generalization when compositionally related samples are shared across training and test sets. However, the PM-PB model remained consistently superior to the purely data-driven baseline under all three validation protocols. This result supports that the advantage of jointly incorporating physical metallurgy descriptors and property-bridging transfer learning is robust to the evaluation protocol.


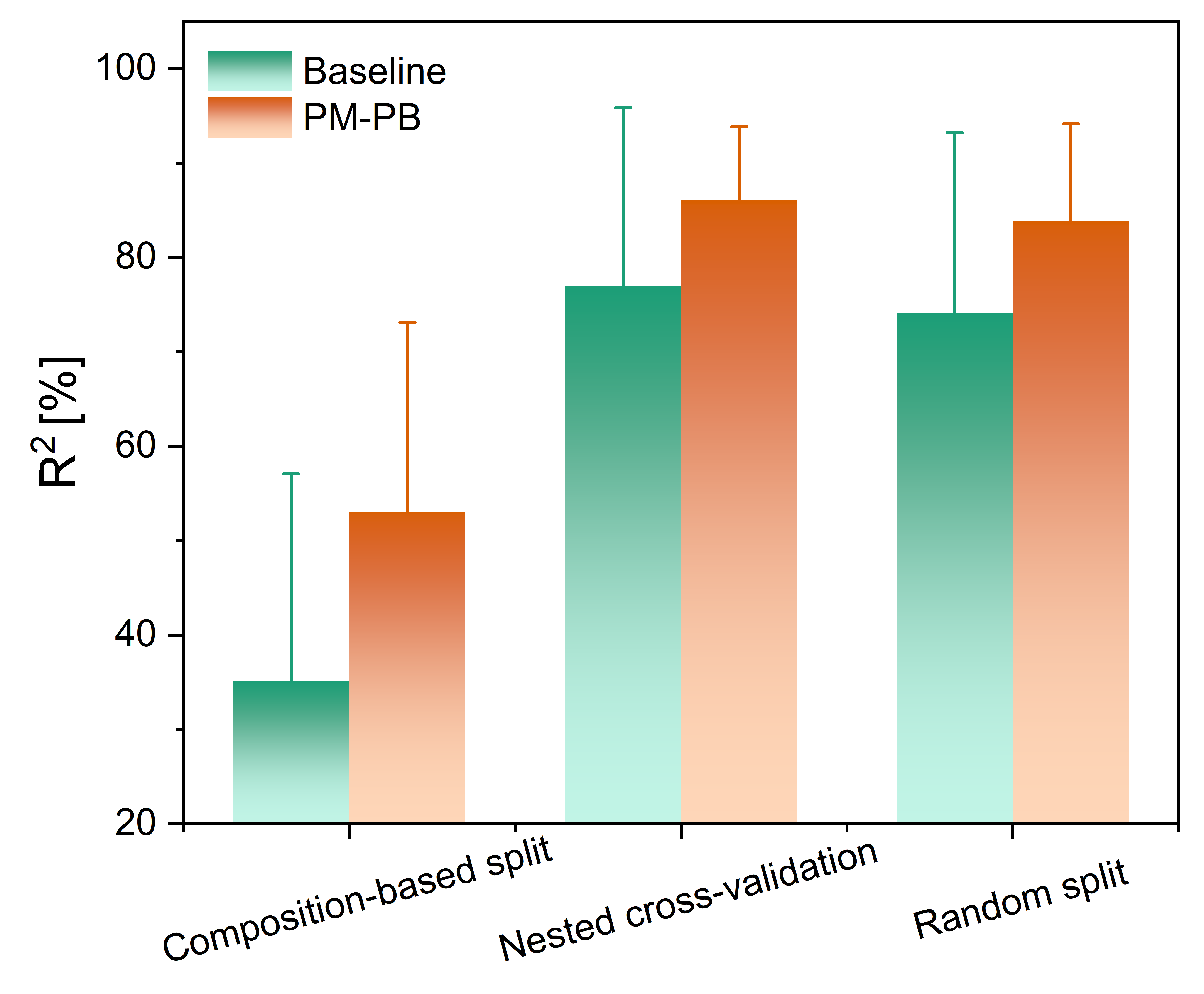


Figure S2. Comparison of UTS prediction performance under different validation protocols for the baseline and PM-PB models.

1. **Source-heterogeneity analysis**

To evaluate potential source-dependent bias in the UTS dataset, a source-heterogeneity analysis was performed for both the purely data-driven baseline model and the PM-PB model. As shown in Figure S3a and b, residuals grouped by data source indicate that inter-source variability is indeed present, which is expected given differences in specimen preparation, testing practice, heat-treatment details, and reporting precision across different studies. However, the deviations are not uniformly directional across all sources, suggesting that source heterogeneity does not manifest as a single systematic offset shared by all external data sources. A direct source-wise comparison between the baseline and PM-PB models further shows that PM-PB achieves lower MAE than the baseline for a substantial fraction of data sources (Figure S3c and e), although not for every individual source. The comparison of source-wise mean residuals (Figure S3d) likewise indicates that PM-PB reduces systematic deviation for several sources, while residual source-dependent variability remains. In addition, the dependence of MAE improvement on source size (Figure S3f) suggests that some of the largest source-wise fluctuations occur in small-source subsets, indicating that limited sample size may amplify apparent source-specific effects. Overall, these results show that hidden source effects cannot be fully excluded, but the predictive advantage of PM-PB is not simply attributable to one favorable data source. In summary, source heterogeneity remains a limitation of the present study. In future work, the incorporation of additional standardized tensile property datasets to augment the existing data will help further enhance the robustness and generalizability of the proposed framework.


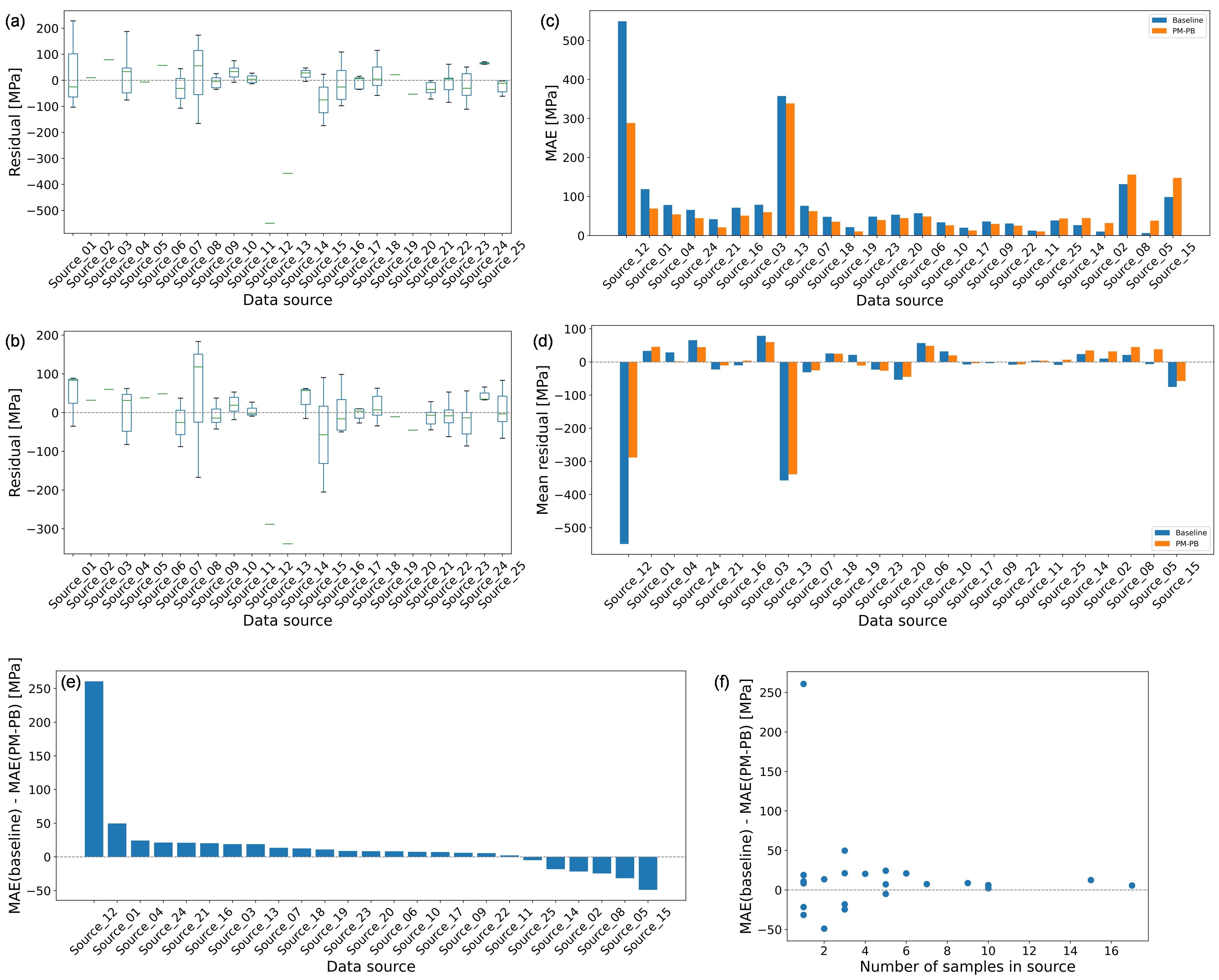


Figure S3. Source-heterogeneity analysis for UTS prediction. (a, b) Residual distributions of the purely data-driven baseline model and the PM-PB model grouped by data source. (c) Source-wise comparison of MAE between the baseline and PM-PB models. (d) Source-wise comparison of mean residual between the baseline and PM-PB models. (e) Source-wise MAE improvement of PM-PB over the baseline, defined as MAE_baseline_-MAE_PM-PB_. (f) Dependence of source-wise MAE improvement on source size.

1. **SHAP analysis for PM-PB**

SHAP analysis was conducted for the final PM-PB models to interpret the contribution of individual input features to UTS and TEL prediction. As shown in Figure S4a and b, the UTS model is mainly influenced by several physical-metallurgy descriptors, particularly Ms_1_, Bs, *f*_M2_, *f*_F_, *f*_B_, and *f*_M1_, together with several key processing and compositional variables. For TEL prediction (Figure S4c and d), important contributors likewise include both physical-metallurgy descriptors and selected process/composition features, with *f*_A1_, Bs, Ms_1_, *f*_F_, and *f*_B_ ranked among the most influential. These results indicate that the PM-PB framework relies strongly on descriptors associated with transformation temperatures, phase evolution, and retained-austenite-related microstructural states, thereby providing additional interpretive support for its physics-guided nature.


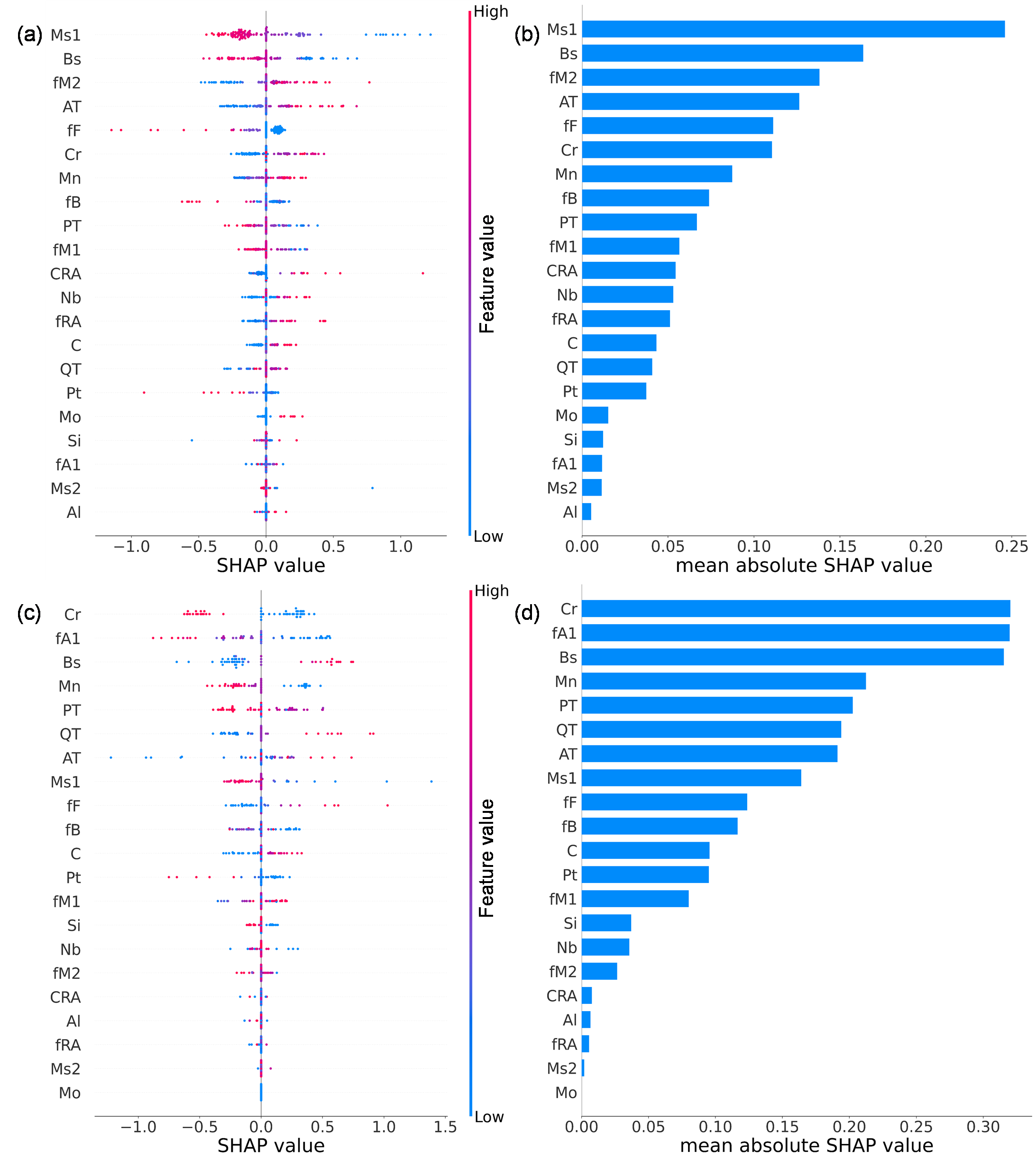


Figure S4. SHAP analysis of the final PM-PB models for UTS and TEL prediction. (a, c) SHAP summary (beeswarm) plots for the final PM-PB models predicting (a) UTS and (c) TEL. Each point represents one sample, and the color indicates the normalized feature value from low to high. The horizontal position denotes the SHAP value, i.e., the contribution of the corresponding feature to the model output. (b, d) Feature-importance ranking based on the mean absolute SHAP value for the final PM-PB models predicting (b) UTS and (d) TEL.

1. **Physical-metallurgy (PM) feature calculation**

To provide physically interpretable inputs for the PB models, ten physical-metallurgy (PM) descriptors were calculated for each alloy–processing condition, namely: Ms_1_ (°C), Bs (°C), *f*_F_, *f*_M1_, *f*_A1_, *f*_B_, *C*_RA_ (wt.%), Ms_2_ (°C), *f*_M2_, and *f*_RA_. These descriptors were designed to represent the dominant transformation pathway in quenching and partitioning (Q&P) steels: austenitization→primary martensitic transformation during quench→possible bainitic transformation during partitioning→carbon partitioning and stabilization of retained austenite→secondary martensite formation during final cooling. The calculation integrates (i) equilibrium phase fractions at the austenitization temperature, (ii) empirical martensite/bainite start temperatures, and (iii) a constrained-carbon-equilibrium with the T_0_ model for the bainitic transformation (CCET) description of carbon redistribution during partitioning.^[1]^

For a given composition (in wt.%) and austenitization temperature (AT), the equilibrium phase fractions at AT were first evaluated thermodynamically using Thermo-Calc. In particular, the austenite fraction at AT, denoted as *f*_A_ (Austenite fraction), and the ferrite fraction at AT (*f*_F_) were obtained from equilibrium calculations. These fractions were used to define an effective carbon content in the austenite available for subsequent transformations. Because a non-negligible fraction of ferrite phase may exist at AT and can sequester a small amount of carbon, the effective austenite carbon concentration was corrected by a carbon-balance adjustment, written as:

$$C_{A}= \frac{C_{0}-C_{F}f_{F}}{f_{A}}$$

where *C*_0_ is the nominal bulk carbon content (wt.%), $f_{F}$ is the ferrite fraction at AT, $f_{A}$ is the austenite fraction at AT, and $C_{F}$ is a small empirical carbon retention factor for the ferrite constituent (0.0218 wt.%). This correction ensures that the subsequent transformation temperatures are governed by the carbon content dissolved in austenite after austenitization.

Using $C_{A}$ and the alloying contents (wt.% Mn, Si, Al, Cr, Mo, Nb), the primary martensite start temperature Ms_1_ and the bainite start temperature Bs were calculated via the empirical relations^[2, 3]^ adopted in this work:

$${Ms}_{1}=\left( 724.5-273.15 \right)+\left( -190.3C_{A}-91.3{C_{A}}^{2} \right)-16.3Mn-4.4Si+\left( -3.5Cr-0.12{Cr}^{2}-0.008{Cr}^{3} \right)-5.9Mo$$

$$Bs=835-198C_{A}-91Mn-15Si-73Cr-87Mo$$

All concentrations are in wt.% and temperatures are in °C. These expressions provide a physically consistent mapping from composition (and effective austenite carbon content) to the onset temperatures of martensitic and bainitic transformations.

During quenching from AT to the quench temperature QT, a fraction of austenite transforms into primary martensite. The primary martensite fraction formed within the austenite constituent was estimated using a Koistinen–Marburger type relation,^[4]^

$f_{M_{1}}^{A}=1-exp[-\alpha({Ms}_{1}-QT)]$, α = 0.011,

and was then converted to a global fraction by scaling with the austenite fraction at AT:

$$f_{M1}=f_{A}\cdot f_{M_{1}}^{A}$$

Accordingly, the remaining austenite fraction after quench is

​$f_{A1}=f_{A}-f_{M1}$

The pair ($f_{M1}$, $f_{A1}$) therefore quantifies the phase state immediately after quenching, before partitioning begins, while consistently accounting for incomplete austenitization (i.e., $f_{A}$＜1).

Partitioning at temperature PT was treated by explicitly considering the competition between bainitic transformation and carbon partitioning, under the framework of constrained carbon equilibrium theory with T_0_ (CCET).^[1]^ In CCE, only carbon is assumed to redistribute during partitioning, while substitutional alloying elements remain effectively immobile on the partitioning time scale. Consequently, austenite enrichment is bounded by a thermodynamic constraint rather than full multi-component equilibrium. In the present implementation, the maximum attainable carbon level in austenite during partitioning was determined using the T_0_ condition, i.e., the carbon concentration at which austenite and ferrite (or martensite) of identical composition have equal Gibbs free energy. Denoting this limiting carbon concentration as *C*_T0_ (PT), the partitioning process is thermodynamically restricted such that the austenite carbon content does not exceed *C*_T0_ at the given PT.

When Bs > PT, bainitic transformation during partitioning is thermodynamically feasible and was included. A bainite fraction *f*_B_ was estimated from carbon mass balance under the CCET constraint, assuming bainite has a characteristic carbon content *C*_B_ (taken as 0.03 wt.% in this work), while the remaining austenite after bainite formation approaches the T_0_-limited carbon level *C*_T0_. Carbon conservation gives

$$C_{A}=f_{A_{1}}^{A}C_{T0}+f_{B}^{A}C_{B}+f_{M_{1}}^{A}C_{M_{1}}$$

Where $f_{A_{1}}^{A}$ and $f_{B}^{A}$ are fractions within the austenite constituent, and $C_{M1}$ represents the carbon trapped in primary martensite. Under the CCET assumption, substitutional diffusion is neglected and the dominant redistribution is between martensite/austenite, with bainite acting as a competing sink for austenite and a low-carbon product phase. The resulting bainite fraction *f*_B_ (global) and the post-partitioning austenite carbon concentration are obtained consistently from this constrained carbon balance, with non-physical negative solutions clipped to zero. When Bs ≤ PT, bainite formation during partitioning was neglected, and carbon partitioning was treated primarily as enrichment of the remaining austenite.

The carbon concentration in retained austenite, denoted as *C*_RA_ (wt.%), was then calculated as the carbon content of the austenite remaining after the partitioning step, following CCET-limited redistribution and accounting for the fractions of martensite and bainite already formed. This *C*_RA_ directly governs the stability of austenite during the subsequent cooling step. Based on *C*_RA_ and alloying contents, the secondary martensite start temperature Ms_2_ was recalculated using the same empirical Ms relation as above, but replacing $C_{A}$ by *C*_RA_.

Finally, upon cooling from PT to room temperature, a fraction of the stabilized austenite transforms into secondary martensite, estimated again using a Koistinen–Marburger type expression evaluated between Ms_2_ and room temperature (taken as 25 °C):

$$f_{M_{2}}^{A}=[1-\exp\left( -\alpha\left( {Ms}_{2}-25 \right) \right)]\cdot f_{A_{1}}^{A}$$

The global secondary martensite fraction is then obtained by scaling with *f*_A_, and the final retained austenite fraction f_RA_ is computed by phase-fraction closure:

$$f_{RA}=f_{A}-f_{M_{1}}-f_{B}-f_{M_{2}}$$

Together, Ms_1_, Bs, *f*_F_, *f*_M1_, *f*_A1_, *f*_B_, *C*_RA,_ Ms_2_, *f*_M2_, and *f*_RA_ provide a compact yet mechanistically grounded representation of the Q&P transformation sequence, explicitly embedding (i) incomplete austenitization via f_F_, (ii) primary and secondary martensitic reactions via Ms_1_/Ms_2_ and *f*_M1_/ *f*_M2_, (iii) possible bainitic competition via Bs and *f*_B_, and (iv) CCET-governed carbon redistribution through the T_0_-limited enrichment that determines C_RA_ and thus the final retained austenite stability.

1. **Statistical summary of hardness and tensile property dataset**

**Table S1**. Statistical summary of input and output features in the hardness dataset.

| Data | Features | Minimum | Maximum | Mean | Standard deviation |
| --- | --- | --- | --- | --- | --- |
| Inputs | C [wt.%] | 0.10 | 0.30 | 0.20 | 0.05 |
|  | Mn [wt.%] | 1.80 | 3.20 | 2.76 | 0.38 |
|  | Si [wt.%] | 1.00 | 2.00 | 1.54 | 0.25 |
|  | Al [wt.%] | 0.04 | 0.80 | 0.18 | 0.27 |
|  | Cr [wt.%] | 0.20 | 0.20 | 0.20 | 0.00 |
|  | Mo [wt.%] | 0.00 | 0.00 | 0.00 | 0.00 |
|  | Nb [wt.%] | 0.00 | 0.00 | 0.00 | 0.00 |
|  | AT [°C] | 760 | 900 | 830 | 58 |
|  | QT [°C] | 180 | 230 | 203 | 21 |
|  | PT [°C] | 350 | 450 | 400 | 41 |
|  | Pt [s] | 100 | 100 | 100 | 0 |
|  | Ms_1_/°C | 315.4 | 378.0 | 348.9 | 18.6 |
|  | Bs [°C] | 453.2 | 593.8 | 497.4 | 36.4 |
|  | *f*_F_ | 0.000 | 0.552 | 0.154 | 0.202 |
|  | *f*_M1_ | 0.276 | 0.887 | 0.672 | 0.185 |
|  | *f*_A1_ | 0.064 | 0.323 | 0.173 | 0.056 |
|  | *f*_B_ | 0.000 | 0.102 | 0.010 | 0.023 |
|  | *C*_RA_ [wt.%] | 0.780 | 1.955 | 1.245 | 0.280 |
|  | Ms_2_ [°C] | -306.2 | 193.9 | 16.2 | 117.9 |
|  | *f*_M2_ | 0.000 | 0.235 | 0.055 | 0.064 |
|  | *f*_RA_ | 0.018 | 0.233 | 0.108 | 0.049 |
| Output | Hardness [HRC] | 23.7 | 45.4 | 36.2 | 5.5 |

**Table S2**. Statistical summary of input and output features in the UTS dataset.

| Data | Features | Minimum | Maximum | Mean | Standard deviation |
| --- | --- | --- | --- | --- | --- |
| Inputs | C [wt.%] | 0.19 | 0.30 | 0.23 | 0.04 |
|  | Mn [wt.%] | 0.67 | 5.70 | 2.44 | 1.09 |
|  | Si [wt.%] | 0.08 | 2.00 | 1.53 | 0.16 |
|  | Al [wt.%] | 0.00 | 1.46 | 0.03 | 0.14 |
|  | Cr [wt.%] | 0.00 | 2.00 | 0.58 | 0.75 |
|  | Mo [wt.%] | 0.00 | 0.56 | 0.04 | 0.15 |
|  | Nb [wt.%] | 0.00 | 0.05 | 0.01 | 0.01 |
|  | AT [°C] | 760 | 1000 | 871 | 58 |
|  | QT [°C] | 20 | 420 | 252 | 74 |
|  | PT [°C] | 200 | 518 | 424 | 51 |
|  | Pt [s] | 0 | 7200 | 326 | 750 |
|  | Ms_1_/°C | 291.4 | 382.3 | 352.2 | 19.4 |
|  | Bs [°C] | 238.4 | 635.3 | 500.3 | 95.0 |
|  | *f*_F_ | 0.000 | 0.473 | 0.038 | 0.093 |
|  | *f*_M1_ | 0.000 | 0.969 | 0.598 | 0.236 |
|  | *f*_A1_ | 0.031 | 1.000 | 0.365 | 0.209 |
|  | *f*_B_ | 0.000 | 0.813 | 0.147 | 0.196 |
|  | *C*_RA_ [wt.%] | 0.403 | 9.546 | 1.233 | 0.998 |
|  | Ms_2_ [°C] | -9736.3 | 283.9 | -60.8 | 971.3 |
|  | *f*_M2_ | 0.000 | 0.467 | 0.142 | 0.096 |
|  | *f*_RA_ | 0.029 | 0.224 | 0.076 | 0.042 |
| Output | UTS [MPa] | 964.0 | 1849.0 | 1315.8 | 182.9 |

**Table S3**. Statistical summary of input and output features in the TEL dataset.

| Data | Features | Minimum | Maximum | Mean | Standard deviation |
| --- | --- | --- | --- | --- | --- |
| Inputs | C [wt.%] | 0.20 | 0.25 | 0.22 | 0.02 |
|  | Mn [wt.%] | 1.10 | 3.00 | 1.98 | 0.69 |
|  | Si [wt.%] | 1.50 | 1.60 | 1.56 | 0.05 |
|  | Al [wt.%] | 0.00 | 0.04 | 0.02 | 0.02 |
|  | Cr [wt.%] | 0.00 | 2.00 | 0.67 | 0.94 |
|  | Mo [wt.%] | 0.00 | 0.00 | 0.00 | 0.00 |
|  | Nb [wt.%] | 0.00 | 0.03 | 0.02 | 0.01 |
|  | AT [°C] | 760 | 1000 | 864 | 74 |
|  | QT [°C] | 200 | 420 | 295 | 56 |
|  | PT [°C] | 200 | 470 | 418 | 54 |
|  | Pt [s] | 34 | 1800 | 338 | 507 |
|  | Ms_1_/°C | 329.5 | 370.6 | 358.8 | 9.9 |
|  | Bs [°C] | 490.0 | 589.4 | 535.2 | 27.4 |
|  | *f*_F_ | 0.000 | 0.473 | 0.078 | 0.120 |
|  | *f*_M1_ | 0.000 | 0.822 | 0.447 | 0.261 |
|  | *f*_A1_ | 0.178 | 1.000 | 0.475 | 0.243 |
|  | *f*_B_ | 0.000 | 0.813 | 0.253 | 0.244 |
|  | *C*_RA_ [wt.%] | 0.780 | 1.540 | 0.965 | 0.163 |
|  | Ms_2_ [°C] | -110.8 | 200.1 | 139.0 | 63.1 |
|  | *f*_M2_ | 0.000 | 0.263 | 0.156 | 0.062 |
|  | *f*_RA_ | 0.034 | 0.152 | 0.066 | 0.030 |
| Output | TEL [%] | 9.2 | 23.6 | 15.8 | 3.6 |


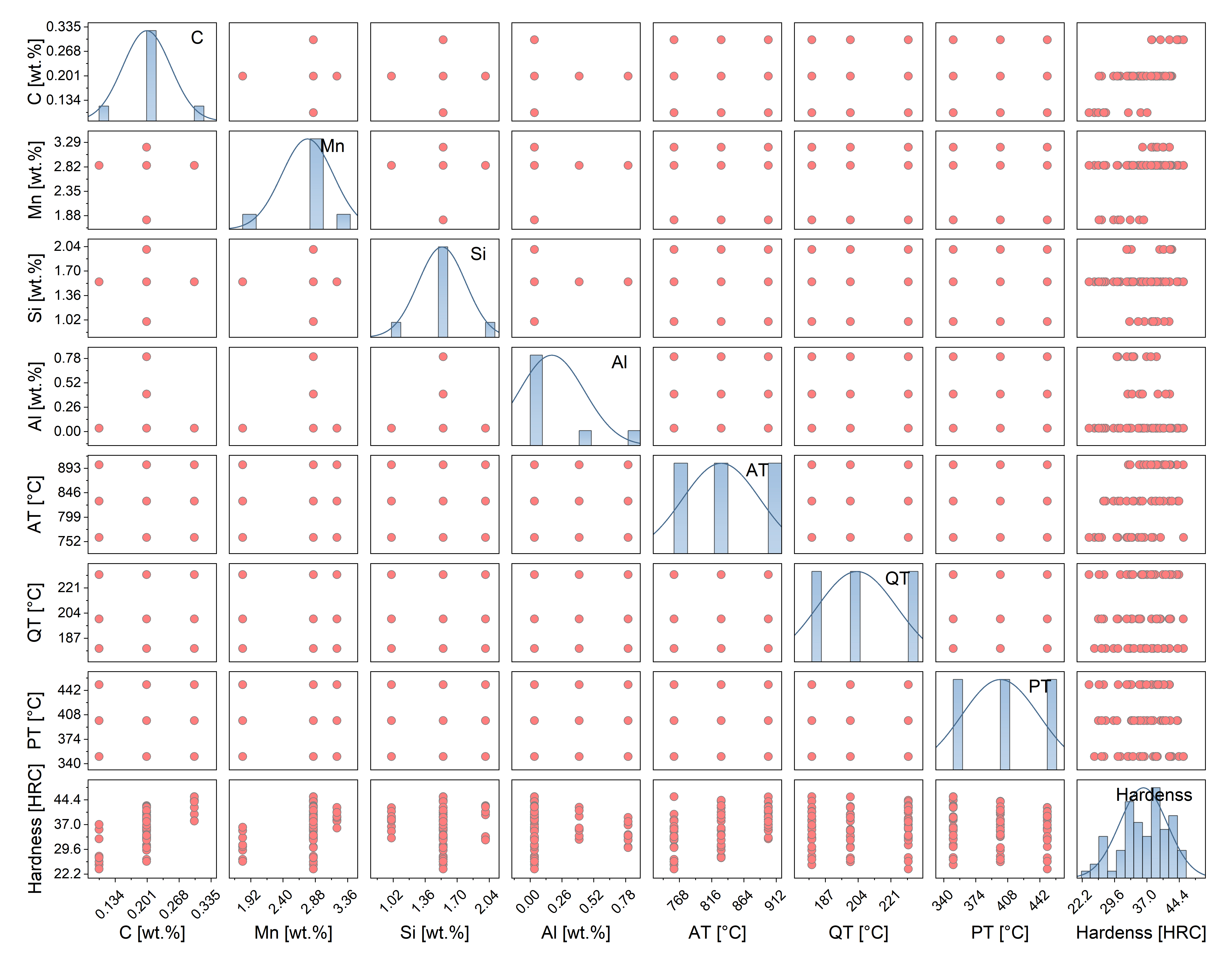


**Figure S5**. Pairwise distributions and correlations between input features (compositional variables and processing parameters) and hardness (HRC).


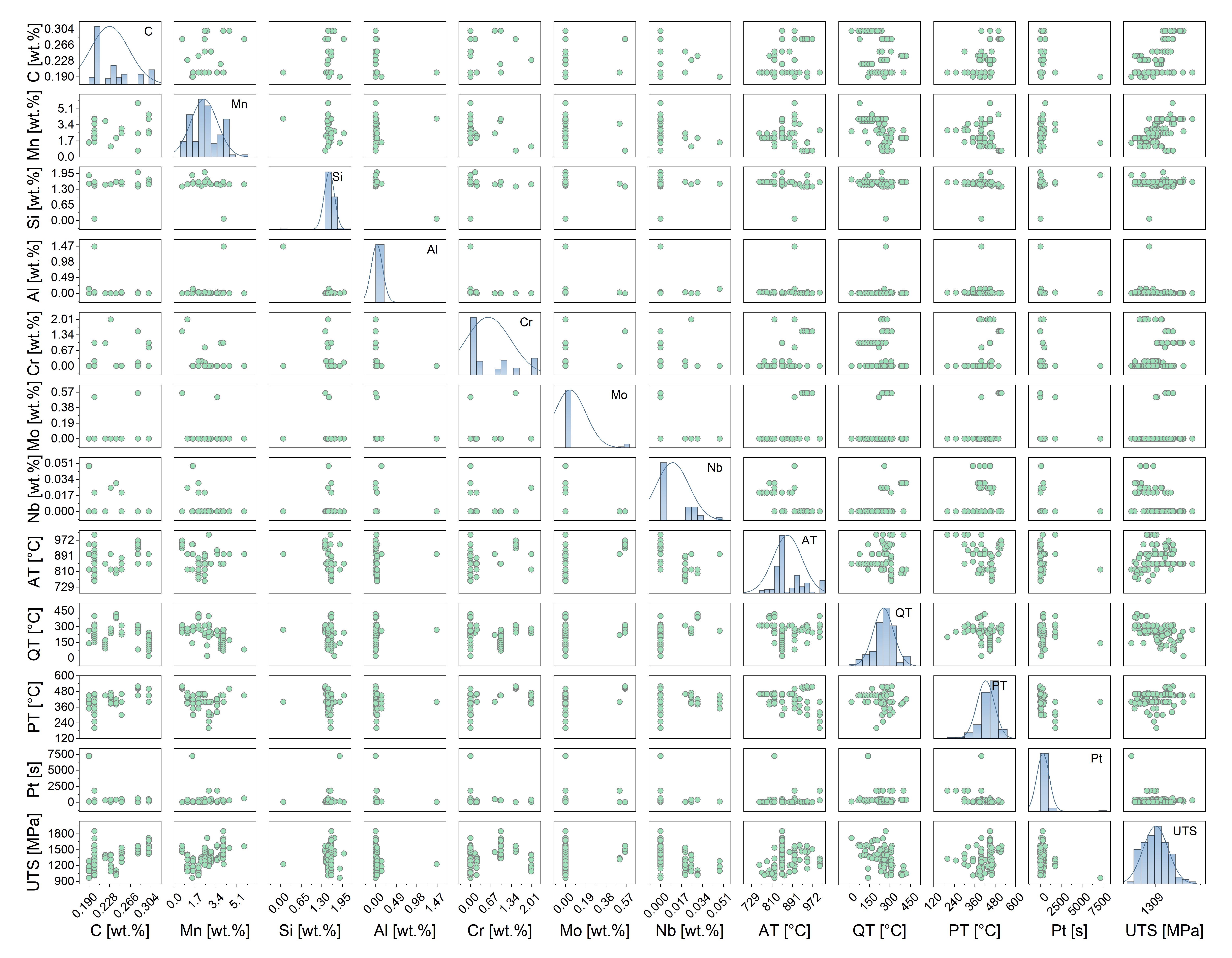


**Figure S6**. Pairwise distributions and correlations between input features (compositional variables and processing parameters) and UTS (MPa).


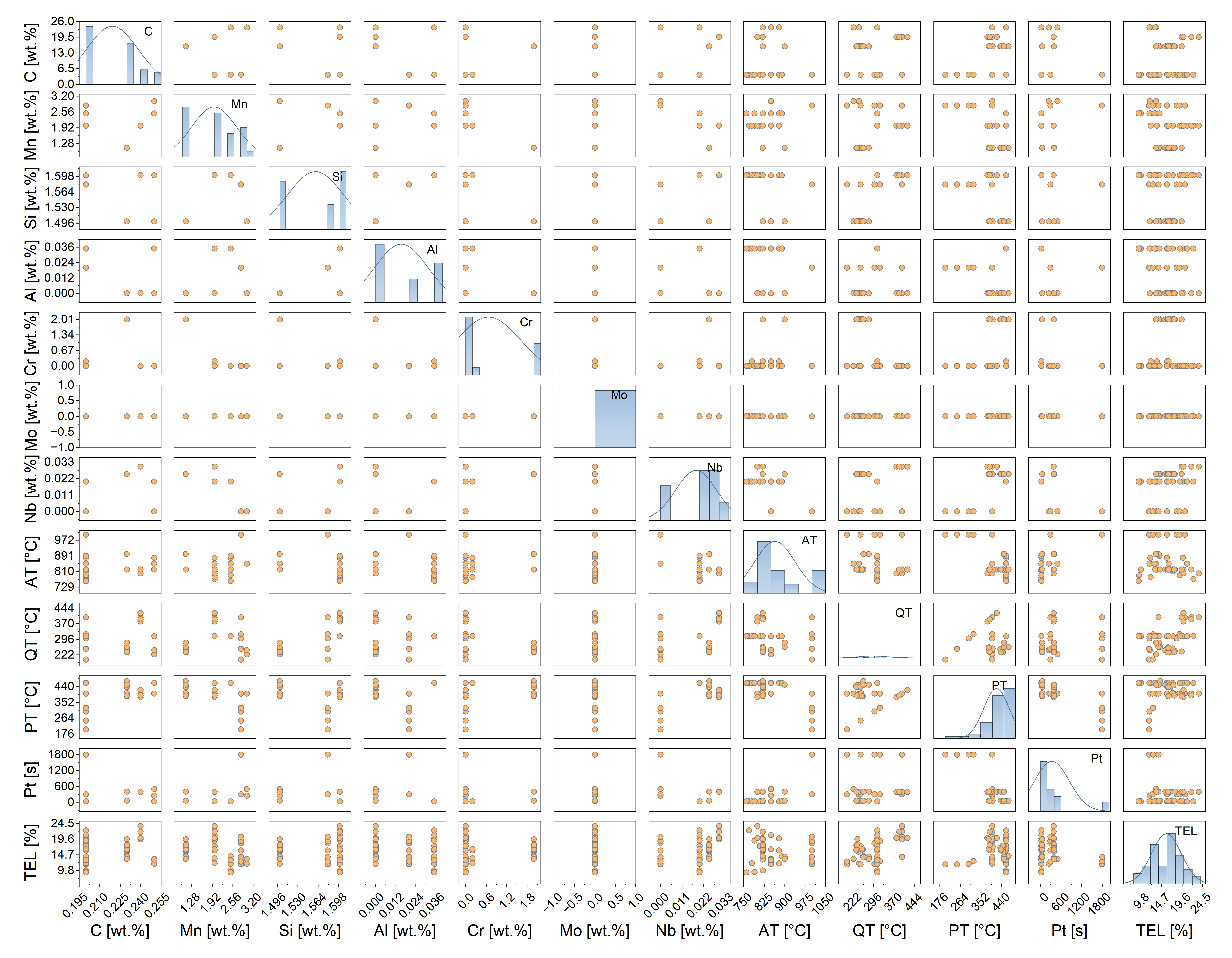


**Figure S7**. Pairwise distributions and correlations between input features (compositional variables and processing parameters) and TEL (%).


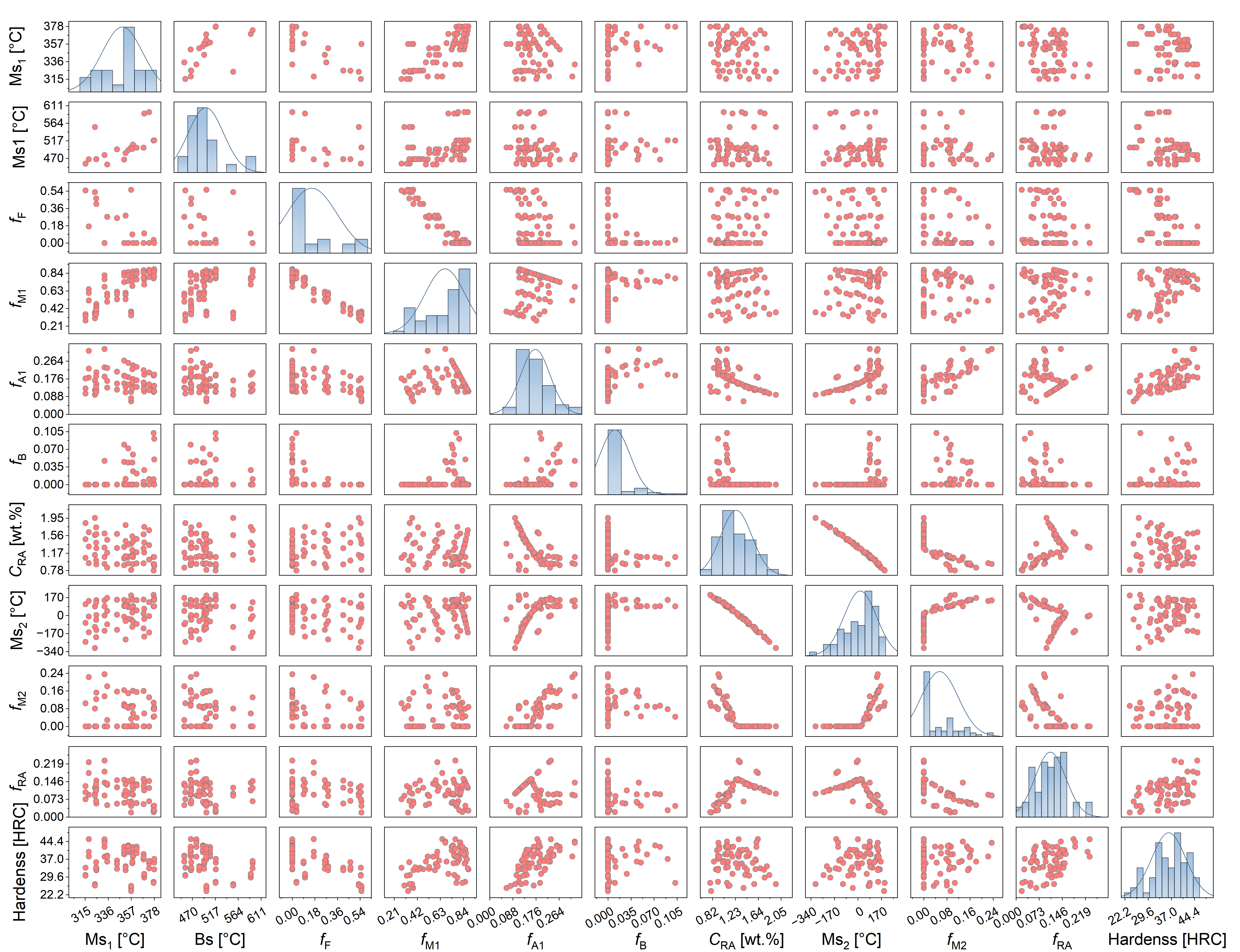


**Figure S8**. Pairwise distributions and correlations between input features (physical-metallurgy parameters) and hardness (HRC).


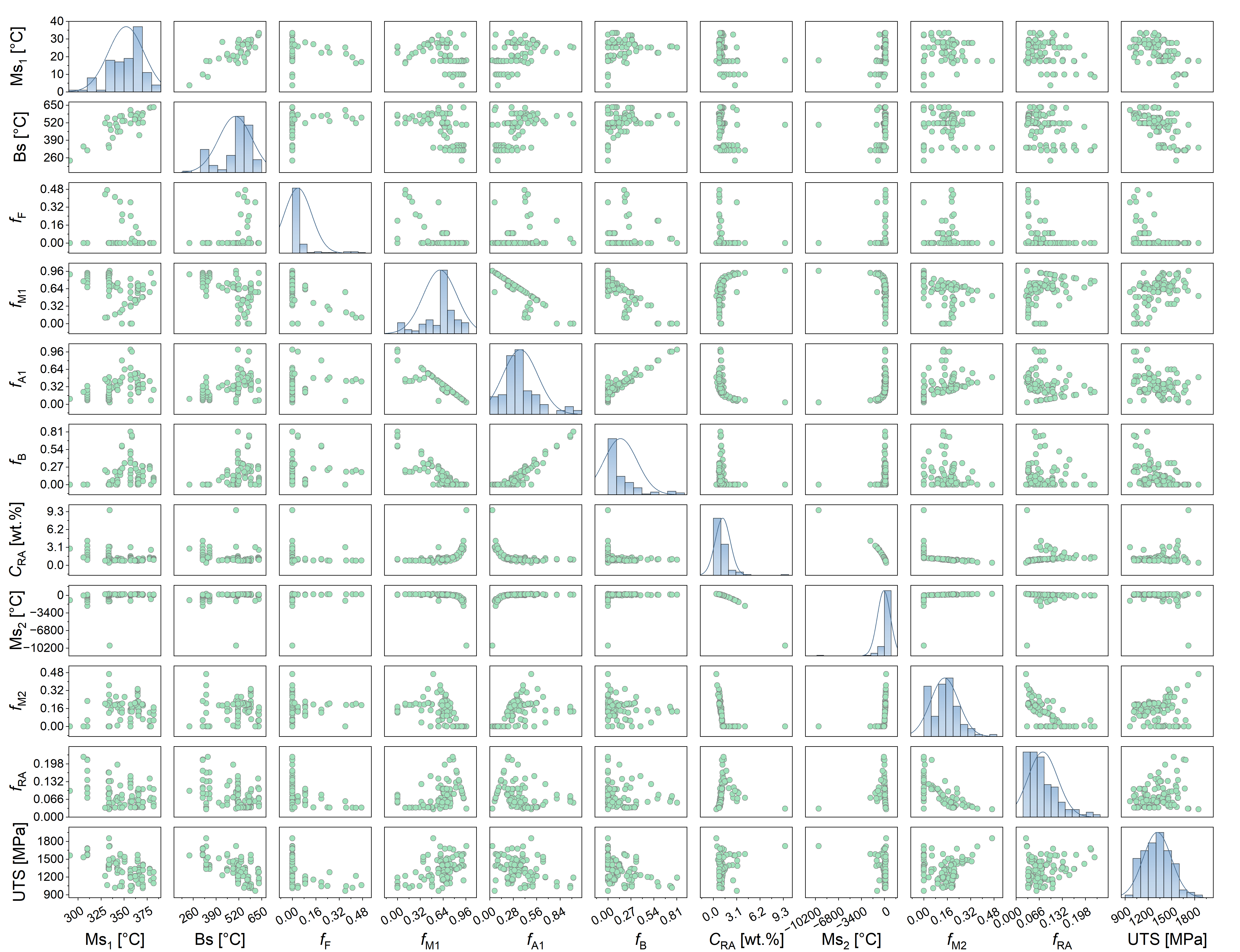


**Figure S9**. Pairwise distributions and correlations between input features (physical-metallurgy parameters) and UTS (MPa).


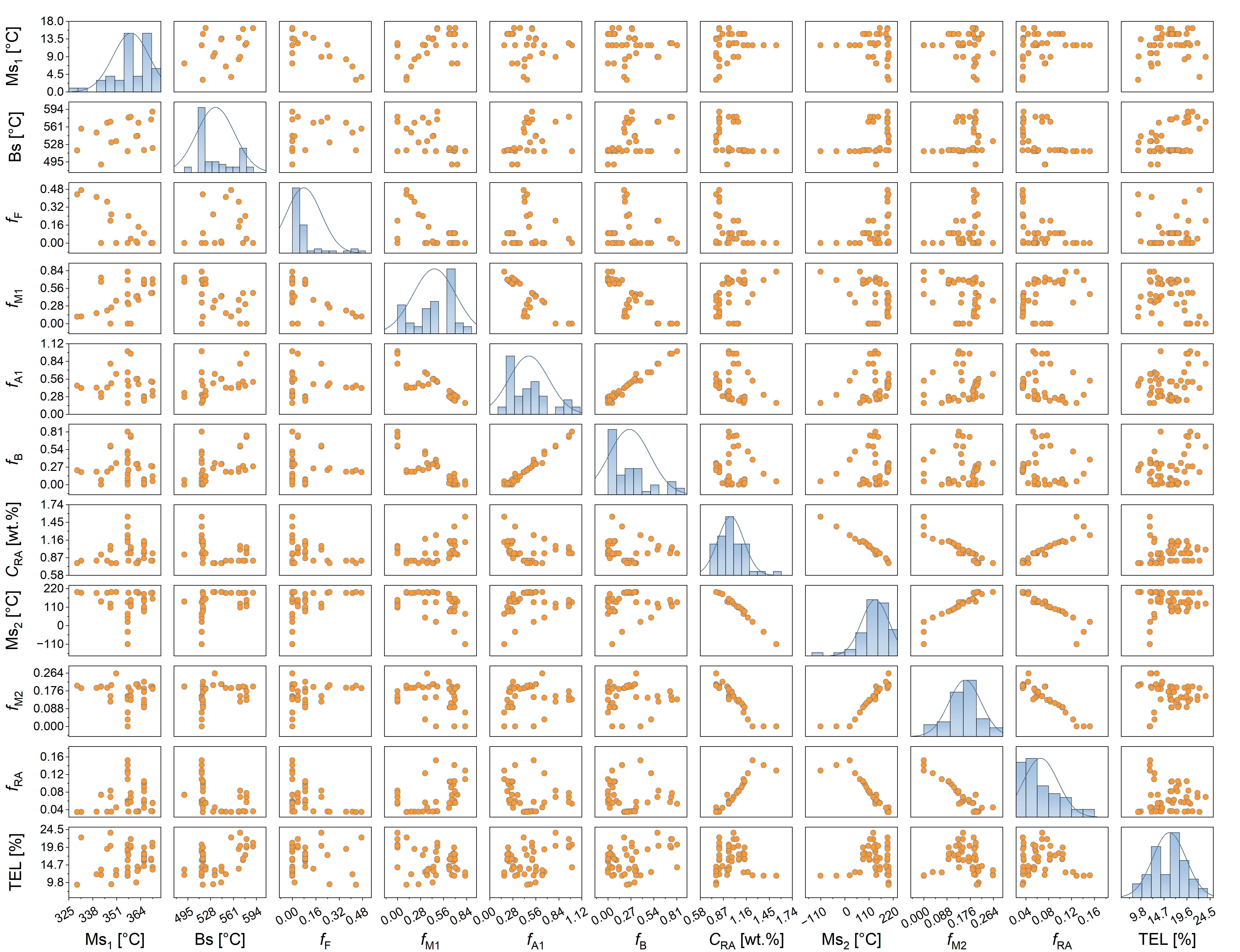


**Figure S10**. Pairwise distributions and correlations between input features (physical-metallurgy parameters) and TEL (%).


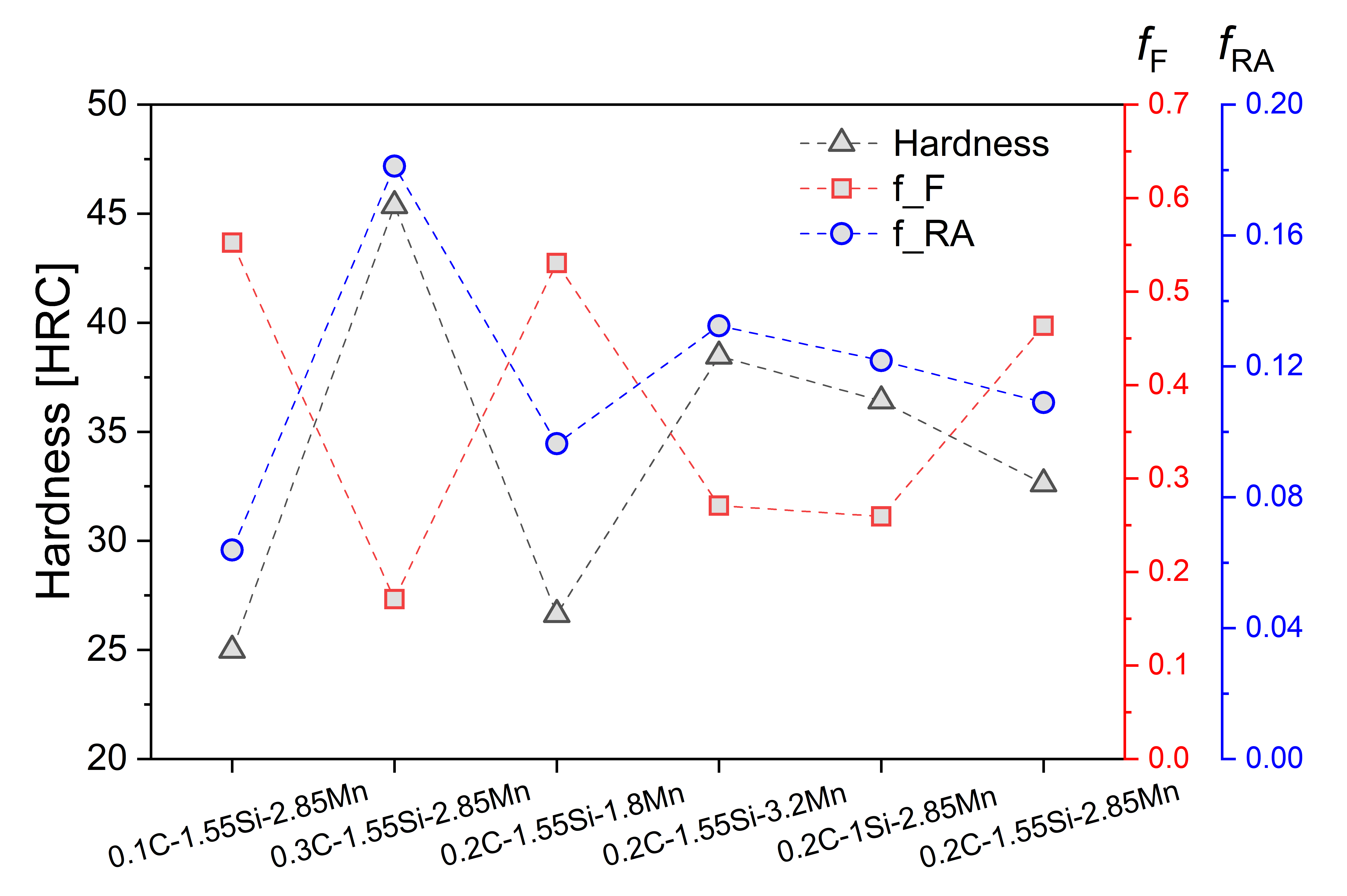


**Figure S11**. Variation of hardness (HRC) with ferrite fraction (*f*_F_) and retained austenite fraction (*f*_RA_) for steels with different chemical compositions.

**Figure S11** presents the variation of hardness (HRC) together with the calculated ferrite fraction (*f*_F_) and retained austenite fraction (*f*_RA_) for representative alloy compositions processed under identical quenching and partitioning conditions. The evolution of hardness exhibits a physically consistent correlation with the calculated phase fractions. An increase in retained austenite fraction is generally accompanied by an increase in hardness (For the six representative alloy compositions, the retained austenite carbon content ranges from approximately 1.38 to 1.96 wt.%), whereas a higher ferrite fraction corresponds to reduced hardness. This behavior reflects the fundamentally different mechanical responses of constituent phases: ferrite is intrinsically softer, while retained austenite is typically associated with higher strength through its stabilization by carbon enrichment and its contribution to transformation-assisted strengthening. The opposite trends of *f*_F_ and *f*_RA_ with respect to hardness are therefore fully consistent with established physical metallurgy principles for multiphase Q&P steels.

In addition, the figure highlights the influence of alloying chemistry on retained austenite stability and hardness under identical processing parameters (AT = 760 °C, QT = 180 °C, PT = 350 °C). Increasing carbon content leads to a pronounced increase in both hardness and retained austenite fraction. For example, when the carbon content increases from 0.1 wt.% to 0.3 wt.% under otherwise identical conditions, the hardness rises from 24.98 HRC to 45.37 HRC, while the retained austenite fraction increases from 6.4% to 18.1%. This trend arises from the dual role of carbon in strengthening martensite and stabilizing austenite by significantly lowering the Ms and Bs temperatures, thereby suppressing complete martensitic transformation during quenching.

Similarly, manganese, as a strong austenite-stabilizing element, exerts a notable effect on both hardness and retained austenite fraction. At a fixed carbon level of 0.2 wt.% and identical processing conditions, increasing the manganese content from 1.8 wt.% to 3.2 wt.% results in an increase in hardness from 26.62 HRC to 38.46 HRC, accompanied by a substantial rise in retained austenite fraction. This behavior reflects the role of manganese in enhancing hardenability, reducing transformation temperatures, and improving the stability of retained austenite during the partitioning stage. Collectively, these observations demonstrate that the calculated phase fractions respond to compositional variations in a physically meaningful manner, thereby validating the reliability of the physical metallurgy–based parameter calculation used in constructing the dataset.

1. **Feature correlation and feature importance analysis**

**Figure S12** and **S13** present the Pearson correlation coefficients (PCCs) between (i) composition and processing parameters and mechanical properties, and (ii) physical-metallurgy parameters and mechanical properties for the three datasets. Clear and physically reasonable correlations are observed in both cases.

For the composition–processing variables, key alloying elements (e.g., C, Mn, Nb) and heat-treatment parameters (e.g., AT, QT, PT) exhibit noticeable correlations with hardness, ultimate tensile strength (UTS), and total elongation (TEL), reflecting their established roles in governing phase transformation behavior, solid-solution strengthening, and hardenability.

For the PM descriptors, phase fractions (e.g., ferrite, martensite, retained austenite) and transformation temperatures (Ms and Bs) show systematic correlations with mechanical properties. In particular, higher martensite and retained austenite fractions are generally associated with increased hardness and strength, whereas higher ferrite fractions correlate with reduced hardness, consistent with classical physical metallurgy principles.

Overall, the observed correlation patterns confirm that the constructed datasets and the physics-based metallurgical calculations capture meaningful structure–property relationships rather than spurious statistical trends, providing a physically consistent foundation for subsequent machine-learning modeling.


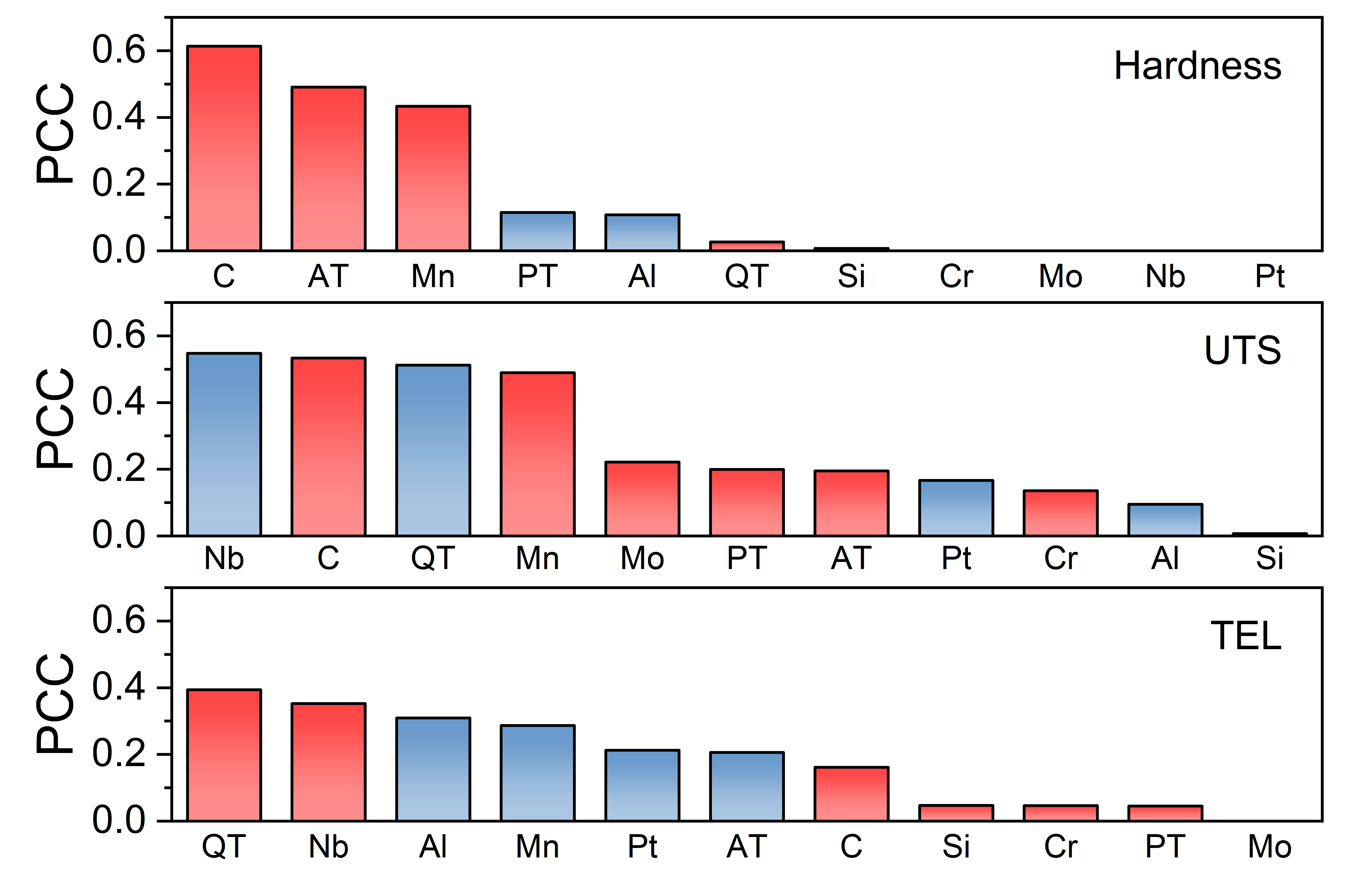


**Figure S12**. Pearson correlation coefficients (PCC) between alloy composition, processing parameters, and mechanical properties (hardness, UTS, and TEL) across the three datasets. Red bars indicate positive correlations, while blue bars indicate negative correlations.


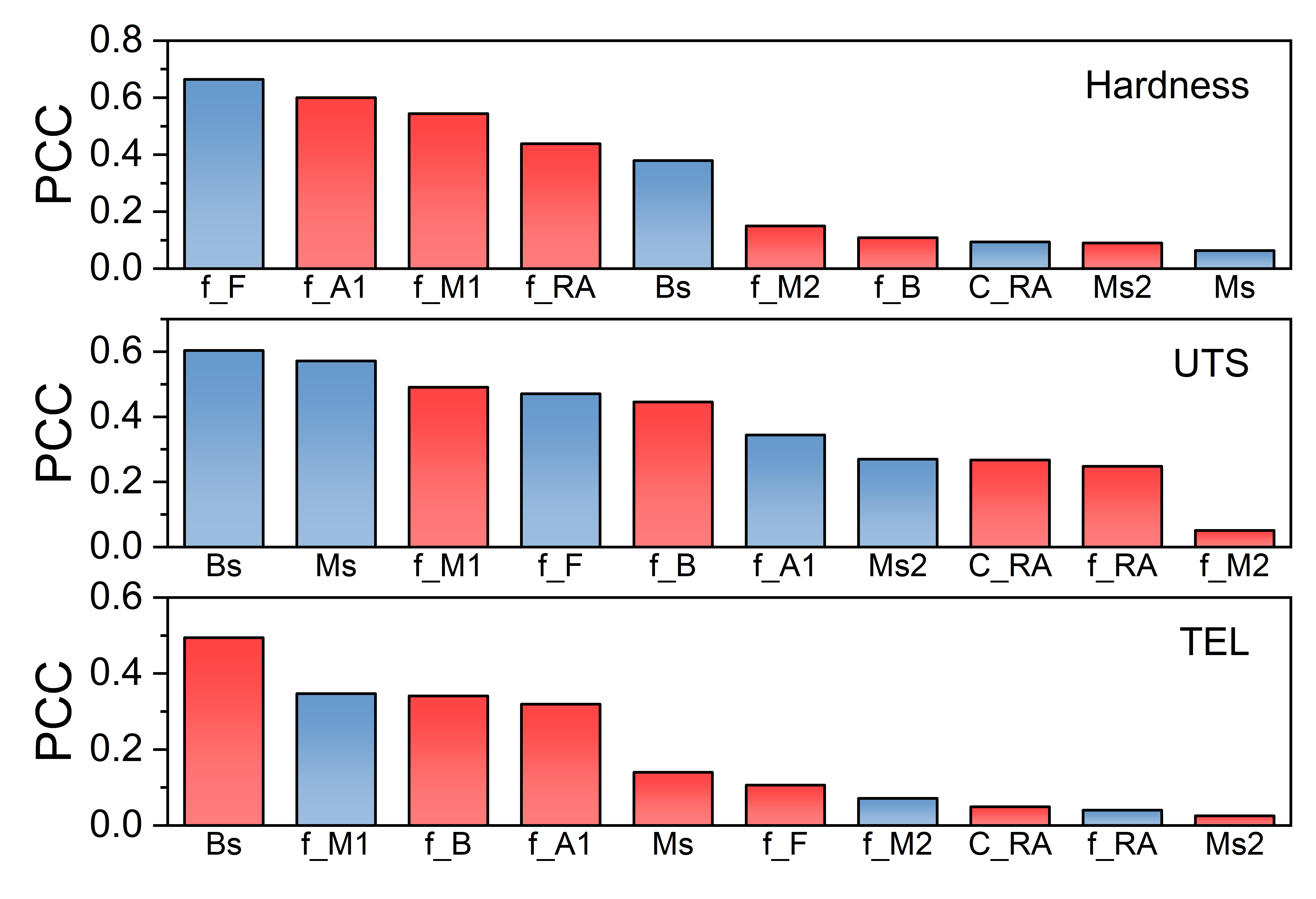


**Figure S13**. Pearson correlation coefficients (PCC) between physical-metallurgy parameters and mechanical properties (hardness, UTS, and TEL) across the three datasets. Red bars indicate positive correlations, while blue bars indicate negative correlations.

The mean decrease accuracy (MDA) analysis^[5]^ based on random forest models (500 repetitions) was employed to evaluate the relative importance of compositional, processing, and physical-metallurgy features for hardness, UTS, and TEL. As shown in **Figure S14**, conventional alloying elements and heat-treatment parameters exhibit clear and physically reasonable contributions to mechanical properties. When PM descriptors are further introduced (**Figure S15**), microstructure-related parameters such as phase fractions, transformation temperatures, and retained austenite stability emerge as dominant contributors, while the relative importance of some original compositional variables is correspondingly reduced. This redistribution of feature importance indicates that PM parameters effectively encode physically meaningful information originally embedded in composition–processing space. Overall, the observed MDA rankings are fully consistent with established physical metallurgy principles, confirming that the calculated PM descriptors provide non-redundant and physically interpretable information for mechanical property prediction.


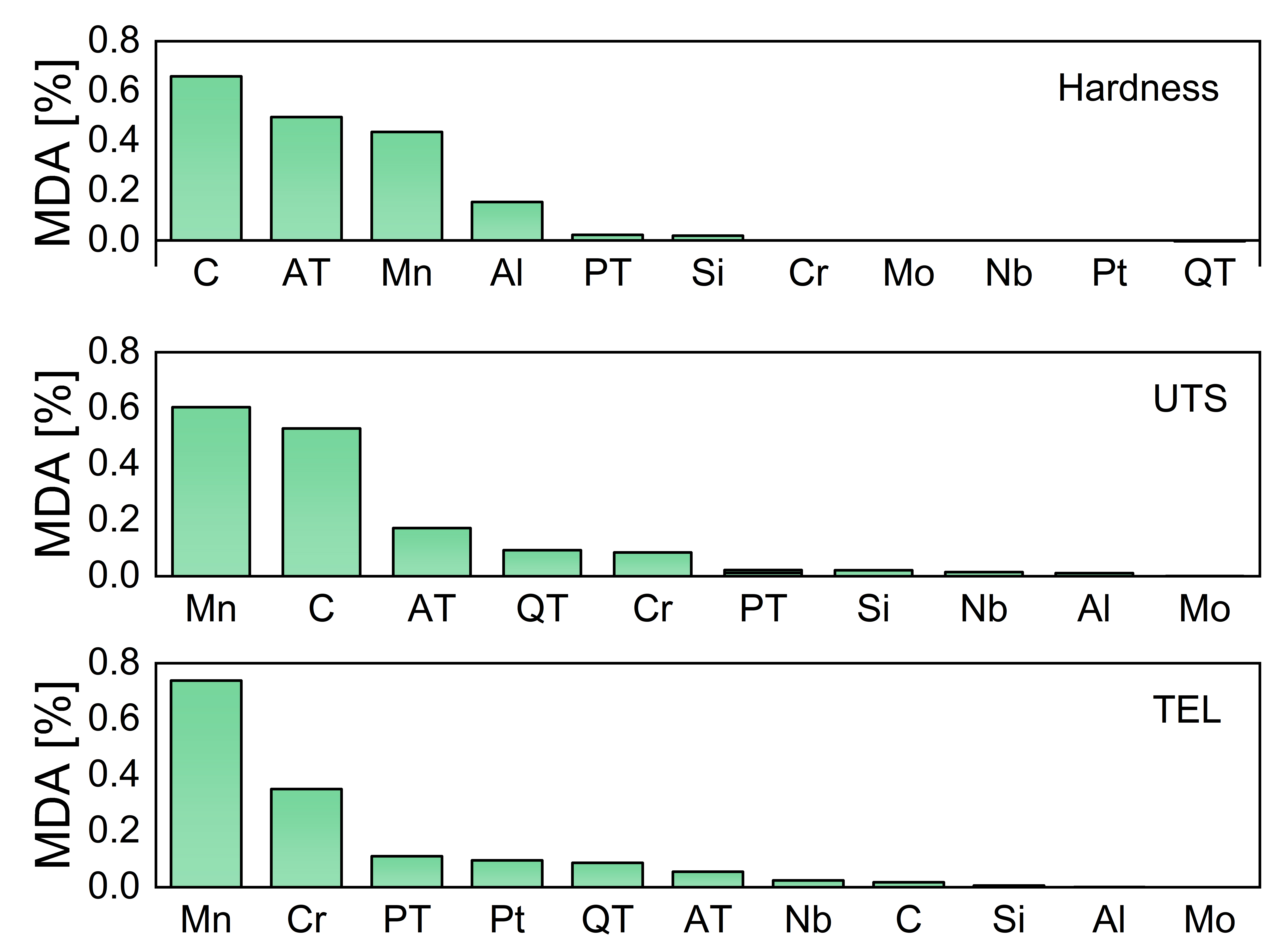


**Figure S14**. Mean decrease accuracy (MDA) rankings of compositional and processing features with respect to hardness, UTS, and TEL, obtained using random forest models with 500 repeated shuffles (Composition and processing parameters are considered as inputs).


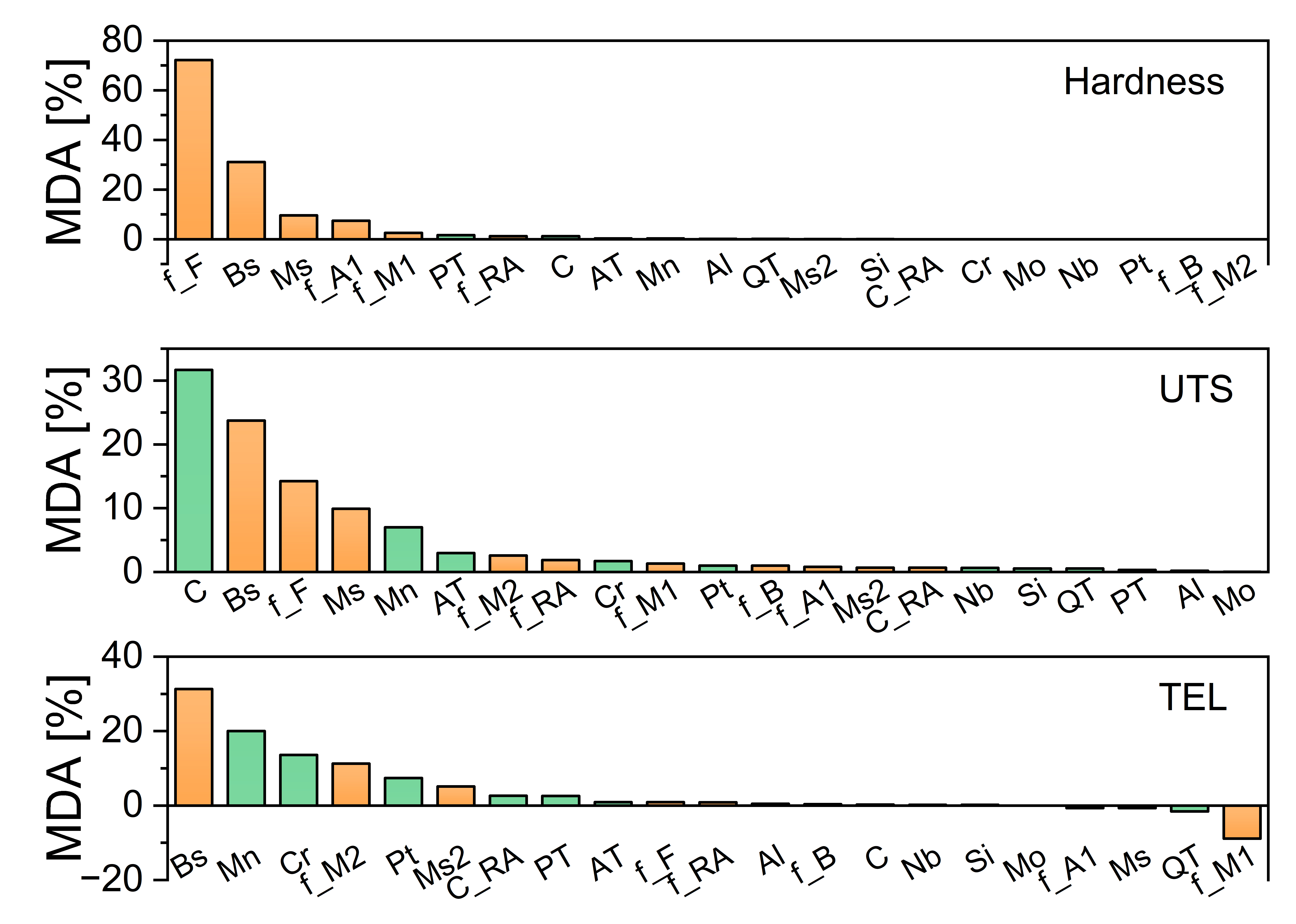


**Figure S15**. Mean decrease accuracy (MDA) rankings of compositional, processing, and physical-metallurgy features with respect to hardness, UTS, and TEL, obtained using random forest models with 500 repeated shuffles.

1. **Property-Bridging Model Details**

All input features were standardized using a z-score normalization. To ensure consistent scaling across datasets, the scaler was fitted on a global Q&P feature pool and then applied to each dataset. Targets (hardness/UTS/TEL) were standardized separately using z-score transform and inverse-transformed after prediction for reporting metrics. Each 21-dimensional standardized feature vector was zero-padded to 25 dimensions and reshaped into a 5×5 feature map. This unified representation allows the same CNN backbone to be used for hardness pretraining and tensile-property transfer.

8.1. Source-CNN for hardness pretraining

The source model was trained on the hardness dataset to learn a generalizable latent representation of composition-processing-PM descriptors. The network uses two convolutional layers followed by fully connected layers and dropout regularization. The model checkpoint with the lowest validation MAE was retained.

**Table S4**. Source-CNN architecture (hardness pretraining).

| Block | Layer | Shape |
| --- | --- | --- |
| Input | Feature map | 5×5×1 |
| Conv-1 | Conv2D | 5×5×8 |
| Conv-2 | Conv2D | 5×5×16 |
| Flatten | Flatten | 400 |
| FC-1 | Dense | 32 |
| FC-2 | Dense | 16 |
| Dropout | Dropout | 16 |
| Output | Dense | 1 |

Training details (Source-CNN): the model was trained using the MAE loss with the Adam optimizer (learning rate = 0.001) for 1000 epochs (batch size = 64).

8.2 PB-CNN for tensile prediction

For tensile-property prediction, a PB transfer network was constructed by combining a frozen source encoder (from the pretrained Source-CNN) and a trainable target encoder. Specifically, the output of the pretrained Source-CNN at the dropout layer was extracted as a transferable embedding and kept non-trainable. A lightweight dense projection was appended to form the source branch output. In parallel, the target branch learns task-specific corrections using a compact CNN. The two branch features are concatenated and mapped to the tensile output by shallow fully connected layers.

**Table S5**. PB-CNN architecture for UTS/TEL (property-bridging transfer).

| Block | Layer | Shape | Trainable |
| --- | --- | --- | --- |
| Input | Feature map | 5×5×1 | - |
| Source branch | Pretrained Source-CNN (to dropout) | 16-d embedding | No |
|  | Dense | 16 | Yes |
| Target branch | Conv2D | 5×5×8 | Yes |
|  | Conv2D | 5×5×16 | Yes |
|  | Flatten | 400 | - |
|  | Dense | 16 | Yes |
|  | Dense | 16 | Yes |
| Fusion | Concatenate | 32 | - |
| Head | Dense | 16 | Yes |
|  | Dense | 8 | Yes |
|  | Dropout | 8 | Yes |
| Output | Dense | 1 | Yes |

Training details (PB-CNN): the model was trained using the MAE loss with the Adam optimizer (learning rate = 0.001) for 2000 epochs (batch size = 64).

8.3. Baseline CNN for tensile prediction

The network uses two convolutional layers followed by fully connected layers and dropout regularization. The model checkpoint with the lowest validation MAE was retained.

**Table S6**. Baseline-CNN architecture (tensile prediction).

| Block | Layer | Shape |
| --- | --- | --- |
| Input | Feature map | 5×5×1 |
| Conv-1 | Conv2D | 5×5×8 |
| Conv-2 | Conv2D | 5×5×16 |
| Flatten | Flatten | 400 |
| FC-1 | Dense | 32 |
| FC-2 | Dense | 16 |
| Dropout | Dropout | 16 |
| Output | Dense | 1 |

Training details (Baseline-CNN): the model was trained using the MAE loss with the Adam optimizer (learning rate = 0.001) for 2000 epochs (batch size = 64).

1. **Rational selection of physics-based metallurgical feature dimensionality**

To determine a reasonable number of physics-based metallurgical (PM) descriptors, random forest models were constructed by incrementally adding PM features and evaluating the corresponding prediction performance. As shown in **Figure S16**, the introduction of PM features leads to a clear increase in R² and a concurrent reduction in MAE at the early stage, indicating the effectiveness of physically informed descriptors. When more PM features are included, the performance gain gradually saturates, and no obvious degradation is observed even with the full set of 10 PM features. This behavior suggests that the PM descriptors are mutually complementary rather than redundant, and supports the adoption of the complete PM feature set in subsequent machine-learning models.


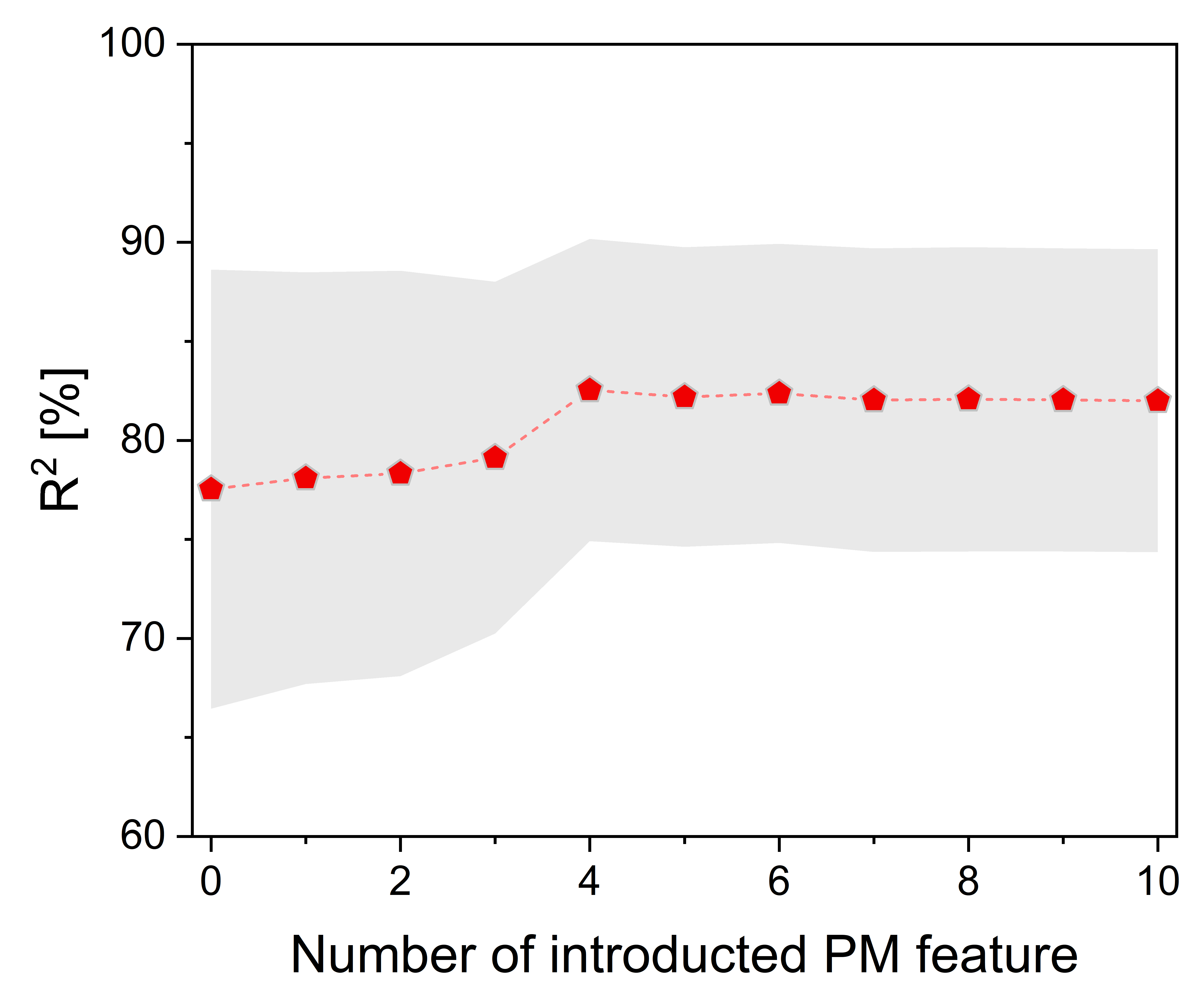

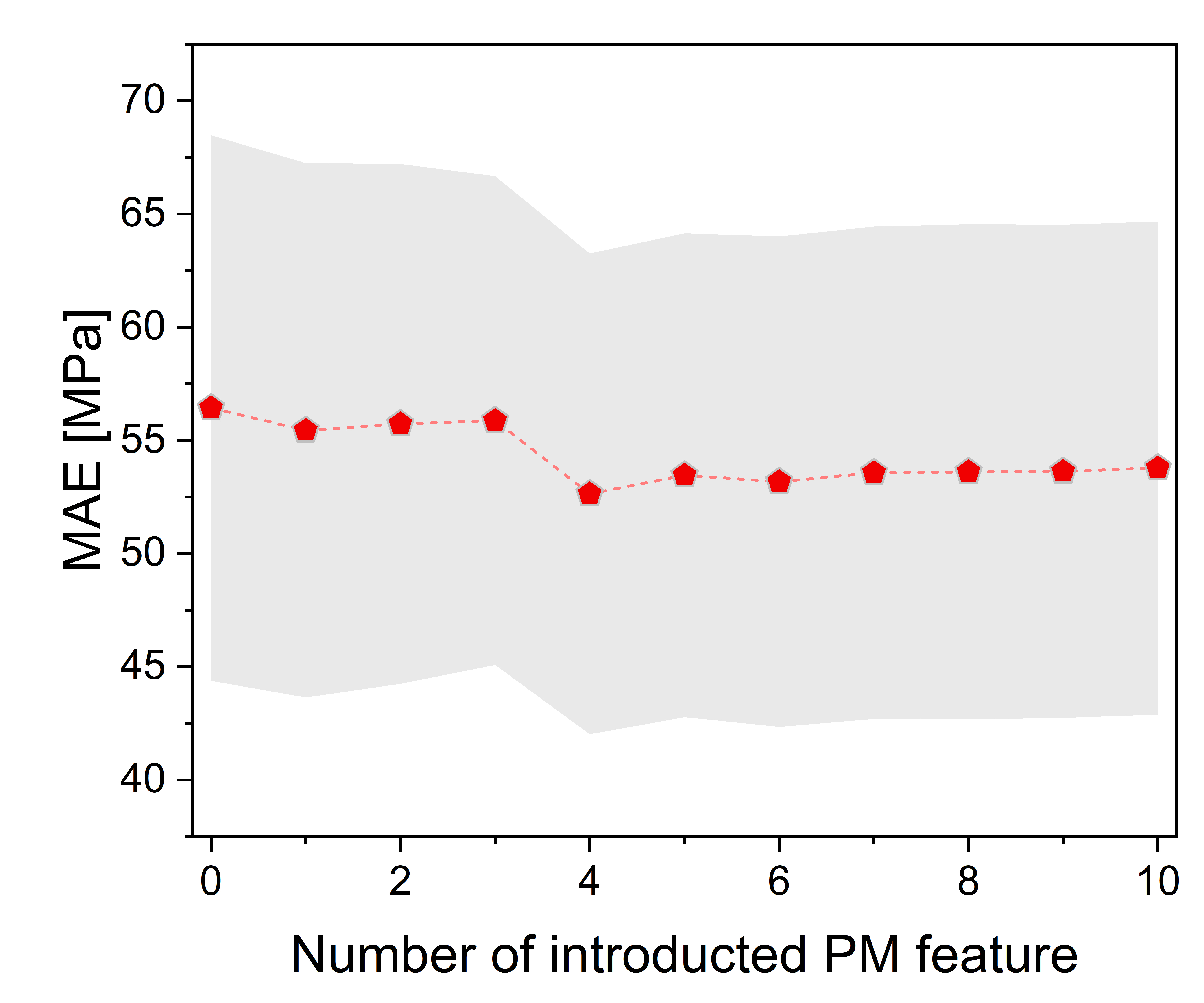


(a)

(b)

**Figure S16**. Effect of the number of introduced physical-metallurgy features on the predictive performance of the UTS model evaluated by random forest. (a) R^2^ and (b) MAE as functions of the number of introduced PM features. The red symbols represent the mean performance over repeated random forest evaluations, while the shaded regions indicate the corresponding variation ranges.

1. **Comparison of different baselines**

**Figure S17** summarizes the performance of the original purely data-driven baseline and additional benchmark models, including XGB, RF, GBR, SVR, and MLP, for UTS and TEL prediction. Although some conventional models show competitive performance in individual tasks, PM-PB still achieves the best overall predictive accuracy and stability.


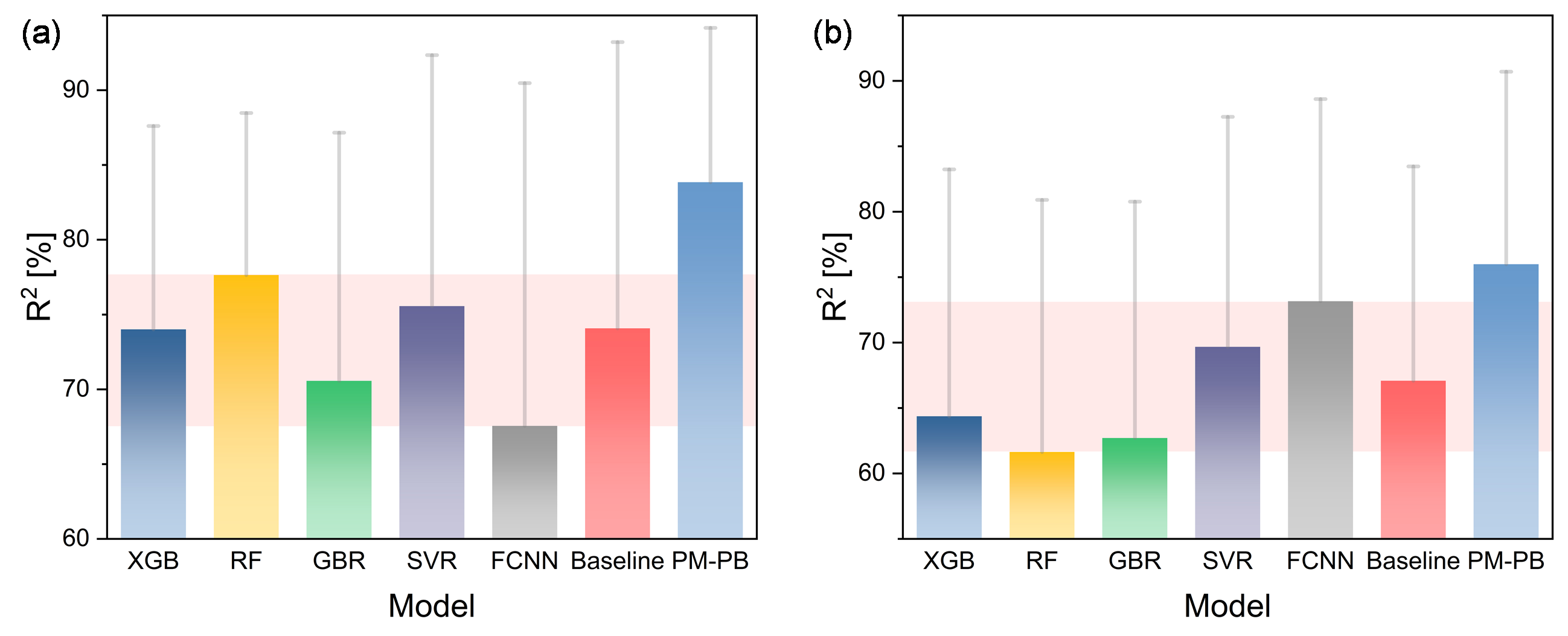


**Figure S17**. Comparative performance of the baseline and additional benchmark models for (a) UTS and (b) TEL prediction under repeated random splits.

**References**

[1] Y. Li, S. Chen, C. Wang, D.S. Martín, W. Xu, Modeling retained austenite in Q&P steels accounting for the bainitic transformation and correction of its mismatch on optimal conditions, Acta Mater. 188 (2020) 528-538.

[2] Y. Li, C. Wang, Y. Zhang, Y. Zhang, L. Wang, Y. Li, W. Xu, Thermodynamically informed graph for interpretable and extensible machine learning: Martensite start temperature prediction, Calphad: Computer Coupling of Phase Diagrams and Thermochemistry 85 (2024).

[3] S.M.C. Van Bohemen, Modeling Start Curves of Bainite Formation, Metall. Mater. Trans. A 41(2) (2010) 285-296.

[4] D.P. Koistinen, R.E. Marburger, A general equation prescribing the extent of the austenite-martensite transformation in pure iron-carbon alloys and plain carbon steels, Acta Metallurgica 7(1) (1959) 59-60.

[5] X. Wei, S. van der Zwaag, Z. Jia, C. Wang, W. Xu, On the use of transfer modeling to design new steels with excellent rotating bending fatigue resistance even in the case of very small calibration datasets, Acta Mater. 235 (2022) 118103.
